# Effect of *CYP1A2*, *CYP2D6*, and *CYP3A4* Variation on Antipsychotic Treatment Outcomes

**DOI:** 10.3390/ph18060892

**Published:** 2025-06-14

**Authors:** Lauren Varney, Stephen Murtough, Marius Cotic, Rosemary Abidoph, Lian Chan, Noushin Saadullah Khani, Alvin Richards-Belle, Maria Richards-Brown, Daisy Mills, Daniele Panconesi, Yogita Dawda, Parveen Sharma, Chetan Shah, Agostina Secchi, Ramin Nilforooshan, Santosh Mudholkar, Rosie Murdoch, Jazmin Molai, Rebecca Griffiths, Suruthy Senthilkumar, Helen Blake, Steve Lankshear, Jennifer McRoberts, Bethany Pastor, Carmel Thomas, Sabrina Richards, Alison Welfare-Wilson, Sai-Bo Cheung, Rebecca Cox, Anita Chinazam Jibero, Reanne Anad, Rebeka Laczik, Sharif Ghali, Alex J. Berry, Joanna Curwen, Koye Odutoye, Girija Kottalgi, Sally Williams, Solomon Wong, Nithya Anandan, Georgy Pius, Tonye Ajiteru, Victoria Clark, Philip van Driel, Amir Bashir, Samantha Court, Minerva Pawsey, Anna Skowronska, Jessica Woodley, Elvira Bramon

**Affiliations:** 1 Division of Psychiatry, University College London, London W1T 7NF, UK; s.murtough@ucl.ac.uk (S.M.); m.cotic@ucl.ac.uk (M.C.); r.abidoph@ucl.ac.uk (R.A.); noushin.khani.21@ucl.ac.uk (N.S.K.); alvin.richards-belle.21@ucl.ac.uk (A.R.-B.); maria.richards-brown.23@ucl.ac.uk (M.R.-B.); daisy.mills.23@ucl.ac.uk (D.M.); daniele.panconesi.20@ucl.ac.uk (D.P.); 2 Department of Genetics and Genomic Medicine, UCL Great Ormond Street Institute of Child Health, University College London, London WC1N 1DZ, UK; lian.chan.22@ucl.ac.uk; 3 North London NHS Foundation Trust, 4th Floor, East Wing, St Pancras Hospital, 4 St Pancras Way, London NW1 0PE, UK; jazmin.molai2@nhs.net (J.M.); alex.berry6@nhs.net (A.J.B.); joanna.curwen@nhs.net (J.C.);; 4 Central and North West London NHS Foundation Trust, 350 Euston Road, Regent’s Place, London NW1 3AX, UK; yogita.dawda@nhs.net (Y.D.); rebecca.griffiths31@nhs.net (R.G.); s.senthilkumar@nhs.net (S.S.); girijakottalgi@nhs.net (G.K.);; 5 Greater Manchester Mental Health NHS Foundation Trust, Bury New Road, Prestwich, Manchester M25 3BL, UK; parveen.sharma@gmmh.nhs.uk (P.S.); jennifer.mcroberts@gmmh.nhs.uk (J.M.); bethany.pastor@gmmh.nhs.uk (B.P.); carmel.thomas@gmmh.nhs.uk (C.T.); nithya.anandan@gmmh.nhs.uk (N.A.); georgy.pius@gmmh.nhs.uk (G.P.); 6 Hertfordshire Partnership University NHS Foundation Trust, The Colonnades, Beaconsfield Road, Hatfield AL10 8YE, UK; chetanshah@nhs.net (C.S.); sabrina.richards5@nhs.net (S.R.); 7 Kent and Medway NHS and Social Care Partnership Trust, Farm Villa, Hermitage Lane, Maidstone ME16 9PH, UK; a.secchi@nhs.net (A.S.); alison.welfare-wilson@nhs.net (A.W.-W.); victoria.clark11@nhs.net (V.C.); 8 Surrey and Borders Partnership NHS Foundation Trust, 18 Mole Business Park, Randalls Road, Leatherhead, Surrey KT22 7AD, UK; ramin.nilforooshan@sabp.nhs.uk (R.N.); sai-bo.cheung@sabp.nhs.uk (S.-B.C.); 9 West London NHS Trust, 1 Armstrong Way, Southall UB2 4SD, UKreanne.anad@nhs.net (R.A.); r.laczik@nhs.net (R.L.); 10 Berkshire Healthcare NHS Foundation Trust, London House, London Road, Bracknell RG12 2UT, UK; rosie2.murdoch@outlook.com (R.M.); sharif.ghali@berkshire.nhs.uk (S.G.); 11 Essex Partnership University NHS Foundation Trust, The Lodge, Lodge Approach, Runwell, Wickford SS11 7XX, UK; helen.blake5@nhs.net; 12 South West London and St George’s Mental Health NHS Trust, Springfield University Hospital, Trinity Building, 15 Springfield Drive, London SW17 0YF, UK; rebecca.cox@swlstg.nhs.uk (R.C.); anita.jibero@swlstg.nhs.uk (A.C.J.); amir.bashir@swlstg.nhs.uk (A.B.); 13 University College London Hospitals NHS Foundation Trust, 250 Euston Road, London NW1 2PG, UK; 14 Somerset NHS Foundation Trust, The Bridge, Priory Park, Wells BA5 1TJ, UK; philip.vandriel@somersetft.nhs.uk; 15 West Midlands Regional Genetics Laboratory, Birmingham Woman’s and Children’s NHS Foundation Trust, Birmingham B15 2TG, UK; samantha.court2@nhs.net (S.C.); minerva.pawsey@nhs.net (M.P.); anna.skowronska@nhs.net (A.S.); jessica.woodley2@nhs.net (J.W.)

**Keywords:** pharmacogenetics, *CYP1A2*, *CYP2D6*, *CYP3A4*, antipsychotics, psychosis, treatment response

## Abstract

**Background/Objectives:** Antipsychotic treatment response varies considerably between individuals, with one potential reason being genetic variation affecting the cytochrome P450 enzymes that metabolise them. **Methods**: With a diverse sample of 453 participants, we studied the influence of *CYP1A2*, *CYP2D6*, and *CYP3A4* variation on three antipsychotic treatment outcomes: participant-reported adverse antipsychotic drug reactions, health-related quality of life, and the dose of antipsychotic medication prescribed. These measures were taken from the baseline assessment, before a pharmacogenetic intervention was delivered. **Results**: Over half of our sample (62.9%) were carriers of an allele associated with altered metabolism of antipsychotic medications on *CYP2D6* or *CYP3A4*, the two genes with pharmacogenetic guidelines for antipsychotic medications. Ultrarapid CYP2D6 metabolisers reported significantly lower levels of adverse antipsychotic drug reactions than normal CYP2D6 metabolisers (mean difference: −11.1; 95% confidence interval [CI]: −18.9, −3.3; *p* = 0.00575). There was also suggestive evidence of lower quality of life scores in those carrying one (mean difference: −0.0863; 95% CI: −0.1806, 0.0081; *p* = 0.0731) or two copies (mean difference: −0.0803; 95% CI: −0.1734, 0.0129; *p* = 0.0914) of the CYP1A2*30-inducible allele. **Conclusions**: Our findings suggest that even when looking at a small number of cytochrome P450 genes, carrying an allele associated with altered antipsychotic medication metabolism is relatively common. We also found evidence that the CYP genotype can influence antipsychotic treatment outcomes, specifically adverse drug reactions and quality of life scores.

## 1. Introduction

Psychosis refers to a range of symptoms that can cause disruption to an individual’s thoughts and/or perceptions [1,2]. Such symptoms can occur in a range of different mental health disorders, most notably schizophrenia and bipolar disorder [3], and, collectively, the prevalence of psychosis is estimated to be around 3% of adults [4,5]. Antipsychotic medications are a key part of the treatment of psychotic disorders [6,7] and have been demonstrated to have moderate to large effect sizes in reducing symptoms at the population level [8]. However, at the individual level, treatment response is more heterogeneous, with 20–30% of those with schizophrenia not responding to first-line antipsychotic treatment [9,10]. This suggests there is room for improving how these drugs are prescribed.

One potential avenue for improvement is a pharmacogenetic approach, which looks at how an individual’s genetic make-up may influence their response to medications [11]. In its application to antipsychotics, pharmacogenetics typically focuses on a few key cytochrome P450 (CYP) enzymes responsible for metabolism and the genes that code for them [12,13,14]. Pharmacogenetic recommendations are available for a number of antipsychotics from the Food and Drug Administration (FDA) in the USA [15] and the Dutch Pharmacogenetics Working Group (DPWG) in the Netherlands [16], but this currently has not been extended to the National Health Service (NHS) in the United Kingdom (UK). The Pharmacogenomics Knowledgebase (PharmGKB) is a National Institute of Health-funded resource that provides clinical annotations for variant-drug pairs based on prescribing guidelines [17]. These are reviewed and assigned a Level of Evidence, based on the quality and strength of the association. The highest of these levels is Level 1A, for which there is at least one variant-specific prescribing guideline available [18].

For medications metabolised by CYP enzymes, the level of enzyme function is determined by the corresponding CYP gene (i.e., the *CYP2D6* gene affects CYP2D6 enzyme function). Individuals with lower enzyme activity will metabolise medications at a slower rate, while those with higher enzyme activity will break down medications faster [19]. To ease interpretation, this spectrum of activity is typically categorised into different metaboliser status groups, ranging from poor metabolisers (little to no enzyme activity) to intermediate metabolisers (reduced activity), normal/extensive metabolisers (typical levels of activity), and rapid and/or ultrarapid metabolisers (increased enzyme activity).

Clinical evidence is currently limited on the efficacy of pharmacogenetic testing for antipsychotics, largely due to there being only a few clinical trials with modest sample sizes. There is also a lack of evidence of the implementation of pharmacogenetics within a psychiatric setting in the UK, though some evidence is available globally from countries including Spain, Denmark, China, and Canada [20,21,22,23,24,25,26]. Evidence that is available typically focuses on CYP2D6 and suggests that poor metabolisers experience more side effects compared to normal metabolisers [27] and require lower doses to reach therapeutic levels [28], and that factoring in pharmacogenetic recommendations in antipsychotic prescriptions can lead to greater symptom improvement [24]. We report initial analyses of the baseline data from an ongoing cohort study, Pharmacogenetics: Genetics and Environment in Mental Health Study, which is, to the best of our knowledge, the first in the UK to offer a pharmacogenetic test to aid in the prescribing of antipsychotic medications. The protocol is available on the Open Science Framework [29].

The aim of this paper was to determine the effects of pharmacogenetic variation within three key genes (*CYP1A2*, *CYP2D6*, and *CYP3A4*) [16] on treatment outcomes of antipsychotic medications. This was to generate new evidence of the effects of variation in pharmacogenes on patient-reported outcomes and medication dose within a UK setting. Our hypotheses were as follows:

1. CYP enzyme activity will be negatively associated with the number of self-reported adverse drug reactions to antipsychotic medications (e.g., individuals with lower CYP activity will report more adverse reactions);
2. Individuals with an extreme metaboliser status (poor or ultrarapid) will score lower on the quality of life scale than normal metabolisers;
3. CYP enzyme activity will be positively associated with antipsychotic medication dose (e.g., those with higher CYP activity will be on higher doses).

## 2. Results

### 2.1. Sample Description

Overall, the sample consisted of 453 participants taking antipsychotic medication, the demographic characteristics of which are presented in Table 1. The sample represented a diverse population, with a broad range of ages (18–82 years), diverse ethnicity (33% identifying as being from an ethnic minority background), and a relatively even split of male and female participants (54% and 46%, respectively).

As the sample is focused on those taking at least one antipsychotic medication, the majority of the participants had a diagnosis of a psychotic disorder, with 88.3% (*n* = 400) having at least one diagnosis of schizophrenia, bipolar disorder, or another psychotic disorder (e.g., schizoaffective disorder or major depressive disorder with psychotic features). The diagnoses for participants without psychosis included major depressive disorder (without psychotic features), emotionally unstable personality disorder, and obsessive–compulsive disorder.

#### 2.1.1. Pharmacogenetic Variation

The frequency of each metaboliser status in the sample is presented in Table 2 (for CYP2D6, these are the metaboliser statuses after accounting for phenoconversion due to concomitant medication; genotype-based metaboliser status frequencies are presented in Table S2). For CYP2D6 and CYP3A4, the most common metaboliser status was a normal metaboliser, and “extreme” metabolisers (poor or ultrarapid) were rare. For *CYP1A2*, no metaboliser status groups have been created based on the new nomenclature [30,31], but carrying a copy of the inducible CYP1A2*30 allele was common. Details of the alleles included on the genotyping panel are presented in Table S3.

A breakdown of the metaboliser statuses per ethnicity group are presented in Table S4, and the diplotypes identified are presented in Tables S5–S7. The allele/metaboliser status frequencies across the ethnicity groups seen in our sample are similar to the patterns seen in other large samples drawn from general populations across multiple ancestries [32,33].

When looking at just the two genes with PharmGKB Level 1A clinical annotations for antipsychotic medications (*CYP2D6* and *CYP3A4*) [34,35], over half the sample were carriers of at least one non-normal function allele with this level of evidence (*n* = 285, 62.9%; Table 3). The proportion carrying each of the alleles is presented in Table S8.

#### 2.1.2. Adverse Antipsychotic Drug Reactions

The Liverpool University Neuroleptic Side Effects Rating Scale (LUNSERS) [36] was used to measure adverse antipsychotic drug reactions. Due to missing data and certain items not being applicable to all the participants, a scaled score was calculated and used as the outcome (more detail in Appendix A). The average scaled score in our sample was 27.4 (SD = 16.6) and ranged from 0 to 76.4, where higher scores denote greater experience of adverse reactions.

#### 2.1.3. Quality of Life

The ED-5Q-5L [37] is a five-item self-report health-related quality of life scale. Responses across the five items can be combined into a single numerical score [38,39], which was used as the outcome. The scores ranged from −0.22 to 1, with an average EQ-5D-5L value of 0.72 (SD = 0.26) and 75 (16.5%) scoring 1 (the highest possible score; no impairment).

#### 2.1.4. Medication Use

The number of participants on each antipsychotic medication is presented in Table 1. To consolidate doses of different antipsychotic medications into a single value, the doses were calculated as a proportion of the maximum licensed dose, according to the British National Formulary (BNF) [40]. The mean dose was 51.2% of the maximum licensed dose (SD = 33.3%), and the total doses ranged from 1.25% to 166.7%. Overall, 26 (5.7%) participants were classified as being on high-dose antipsychotic treatment (>100% of the maximum licensed dose), and 81 (17.9%) were prescribed two different antipsychotic medications.

### 2.2. Effect of Pharmacogenetic Variation

#### 2.2.1. Effect of Pharmacogenetic Variation on Adverse Antipsychotic Drug Reactions

**Figure 1:**
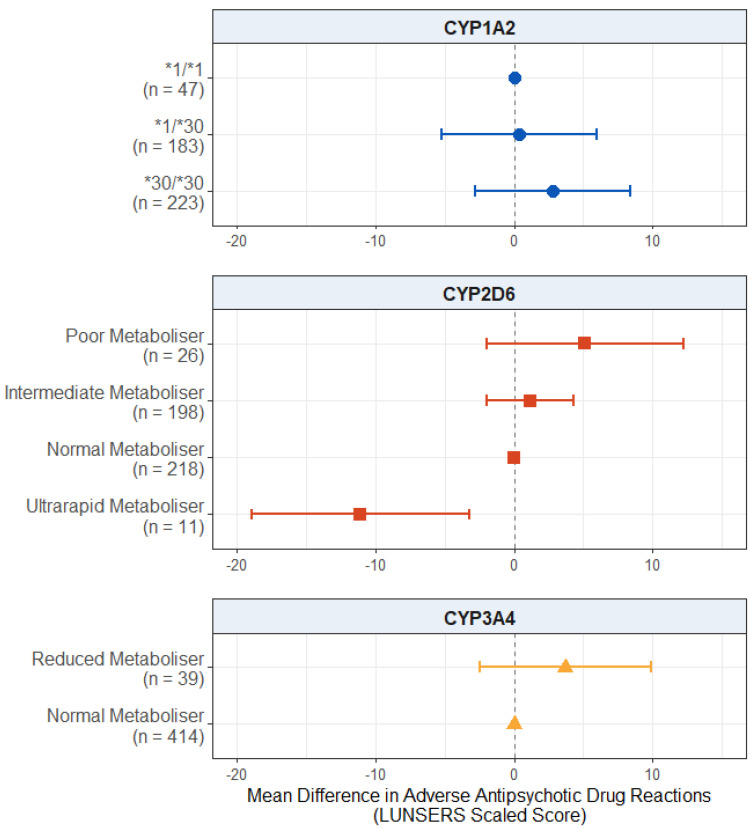
Effect of pharmacogenetic variation in *CYP1A2*, *CYP2D6*, and *CYP3A4* on self-reported adverse drug reactions from antipsychotic medications. Comparing CYP metaboliser phenotypes/diplotypes on self-reported adverse drug reactions from antipsychotic medications, when accounting for covariates. Reference category is CYP1A2*1/1 diplotype, CYP2D6 normal metaboliser status, and CYP3A4 normal metaboliser status. Number of participants (*n*) in each group is shown in brackets. Abbreviations: *n* = number of participants; LUNSERS = Liverpool University Neuroleptic Side Effects Rating Scale.

For the effect of pharmacogenetic variation on adverse antipsychotic drug reactions, displayed in Figure 1, we found a significant negative effect of CYP2D6 ultrarapid metaboliser status. Ultrarapid metabolisers scored, on average, 11.1 points fewer on the LUNSERS scaled score compared to normal metabolisers (95% CI: −18.9, −3.3; *p* = 0.00575). There was no evidence of an effect of either CYP2D6 poor metaboliser status (*p* = 0.158) or intermediate metaboliser status (*p* = 0.466).

We also found no evidence of an effect of *CYP1A2* diplotype (*p* values > 0.333) or reduced CYP3A4 metabolism (*p* = 0.243) on reported adverse antipsychotic drug reactions. The full results are presented in Table S9.

When looking at the associations between CYP metaboliser status/diplotype and adverse antipsychotic drug reactions within each of the LUNSERS domains, the pattern was generally consistent. CYP2D6 ultrarapid metaboliser status was associated with fewer extrapyramidal (mean difference: −12.9; 95% CI: −21.9, −4.0, *p* = 0.00484), autonomic (mean difference: −15.5; 95% CI: −22.5, −8.4, *p* = 0.0000223), psychic (mean difference: −15.6; 95% CI: −28.8, −2.4, *p* = 0.0214), and hormonal (mean difference: −10.1; 95% CI: −18.1, −2.1, *p* = 0.0136) adverse drug reactions. These results are discussed in detail in Appendix B, and the full results are presented in Tables S10–S16.

#### 2.2.2. Effect of Pharmacogenetic Variation on Quality of Life

**Figure 2:**
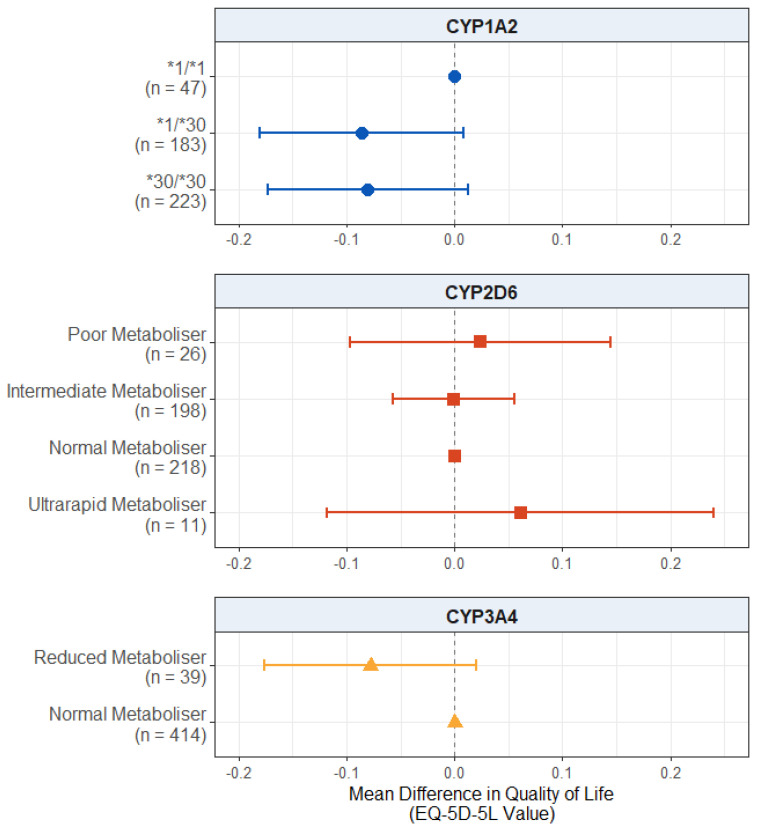
Effect of pharmacogenetic variation in *CYP1A2*, *CYP2D6*, and *CYP3A4* on self-reported health-related quality of life. Comparing CYP metaboliser phenotypes/diplotypes on self-reported adverse drug reactions from antipsychotic medications, when accounting for covariates. Reference category is CYP1A2*1/1 diplotype, CYP2D6 normal metaboliser status, and CYP3A4 normal metaboliser status. Number of participants (*n*) in each group is shown in brackets. Abbreviations: *n* = number of participants.

For the quality of life scores, displayed in Figure 2, we found no significant effect of CYP2D6 metaboliser status group (*p* values > 0.503) or reduced CYP3A4 metabolism (*p* = 0.119). There was also no significant effect of *CYP1A2* diplotype, though the results for both CYP1A2*1/*30 carriers (mean difference: −0.0863; 95% CI: −0.1806, 0.0081; *p* = 0.0731) and CYP1A2*30/*30 carriers (mean difference: −0.0803; 95% CI: −0.1734, 0.0129; *p* = 0.0914) did pass the suggestive threshold of *p* = 0.1, with both groups showing a trend towards lower quality of life scores compared to wildtype CYP1A2*1/*1 carriers. The full results are presented in Table S17.

#### 2.2.3. Effect of Pharmacogenetic Variation on Medication Dose

**Figure 3:**
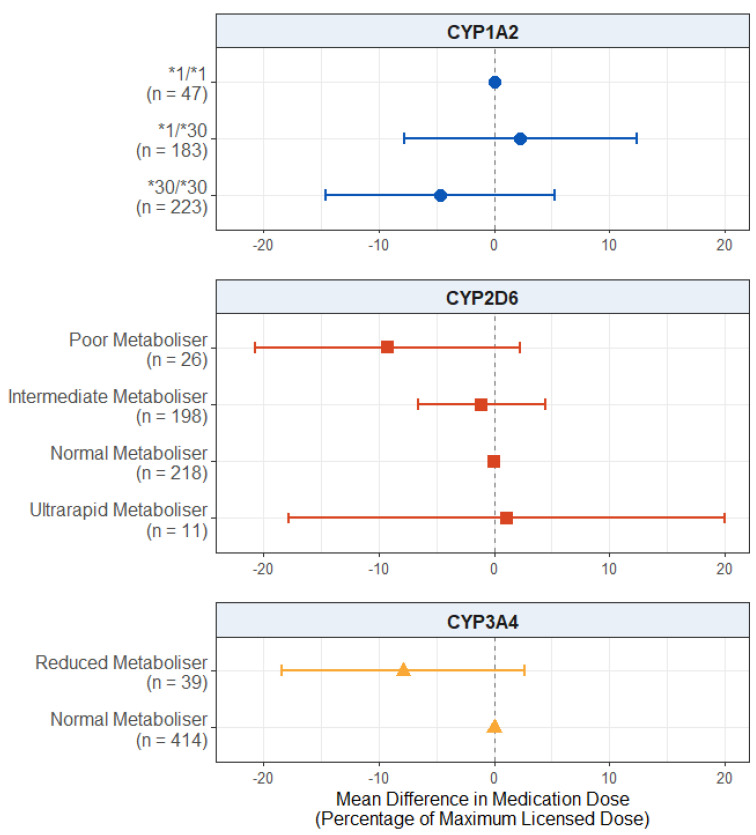
Effect of pharmacogenetic variation in *CYP1A2*, *CYP2D6*, and *CYP3A4* on prescribed antipsychotic medication dose. Comparing CYP metaboliser phenotypes/diplotypes on self-reported adverse drug reactions from antipsychotic medications, when accounting for covariates. Reference category is CYP1A2*1/1 diplotype, CYP2D6 normal metaboliser status, and CYP3A4 normal metaboliser status. Number of participants (*n*) in each group is shown in brackets. Abbreviations: *n* = number of participants.

For the dose of antipsychotic medication prescribed, as displayed in Figure 3, we found no significant effect in the CYP2D6 metaboliser status group (*p* values > 0.115), *CYP1A2* diplotype (*p* values > 0.356), or reduced CYP3A4 metabolism (*p* = 0.142). The full results are presented in Table S18.

### 2.3. Sensitivity Analysis: Psychotic Disorder Diagnosis Only

We carried out a sensitivity analysis including only participants with a diagnosis of psychosis (*n* = 400), due to the potential for different clinical characteristics in those without a diagnosis of psychosis but receiving antipsychotic treatment. The demographic characteristics, and metaboliser status frequencies of this sample are presented in Tables S19 and S20, respectively. The findings remained consistent with our main analysis.

#### 2.3.1. Effect of Pharmacogenetic Variation on Adverse Antipsychotic Drug Reactions in Participants with a Diagnosis of Psychosis

**Figure 4:**
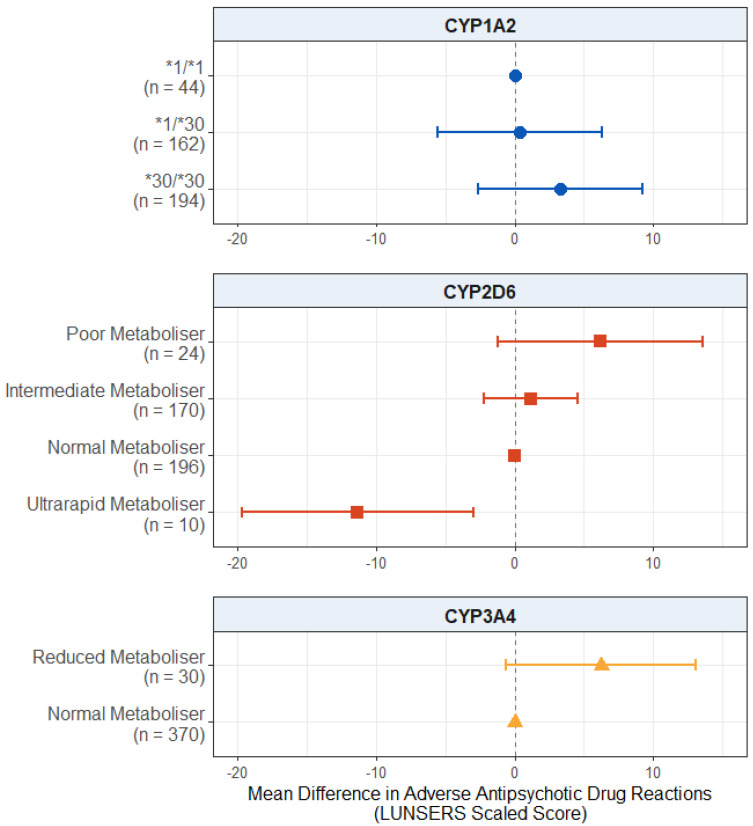
Effect of pharmacogenetic variation in *CYP1A2*, *CYP2D6*, and *CYP3A4* on self-reported adverse drug reactions from antipsychotic medications in participants with a psychosis diagnosis. Comparing CYP metaboliser phenotypes/diplotypes on self-reported adverse drug reactions from antipsychotic medications, when accounting for covariates. Reference category is CYP1A2*1/1 diplotype, CYP2D6 normal metaboliser status, and CYP3A4 normal metaboliser status. Number of participants (*n*) in each group is shown in brackets. Abbreviations: *n* = number of participants; LUNSERS = Liverpool University Neuroleptic Side Effects Rating Scale.

When looking at the effect of pharmacogenetic variation on adverse drug reactions in this subgroup (Figure 4), the effect of CYP2D6 ultrarapid metaboliser status remained significant, with these individuals reporting fewer adverse drug reactions compared to the normal metabolisers (mean difference: −11.3; 95% CI: −19.7, −3.0; *p* = 0.00816). There were no significant effects of CYP2D6 poor metaboliser (*p* = 0.100) or intermediate metaboliser status (*p* = 0.492).

There was no significant effect of the *CYP1A2* diplotype on adverse drug reactions (*p* values > 0.279).

There was no significant effect of reduced CYP3A4 activity, but there was a trend towards significance (mean difference: 6.242; 95% CI: −0.612, 13.095; *p* = 0.0751), where reduced metabolisers reported more adverse drug reactions than normal metabolisers. The full results are presented in Table S21.

#### 2.3.2. Effect of Pharmacogenetic Variation on Quality of Life in Participants with a Diagnosis of Psychosis

**Figure 5:**
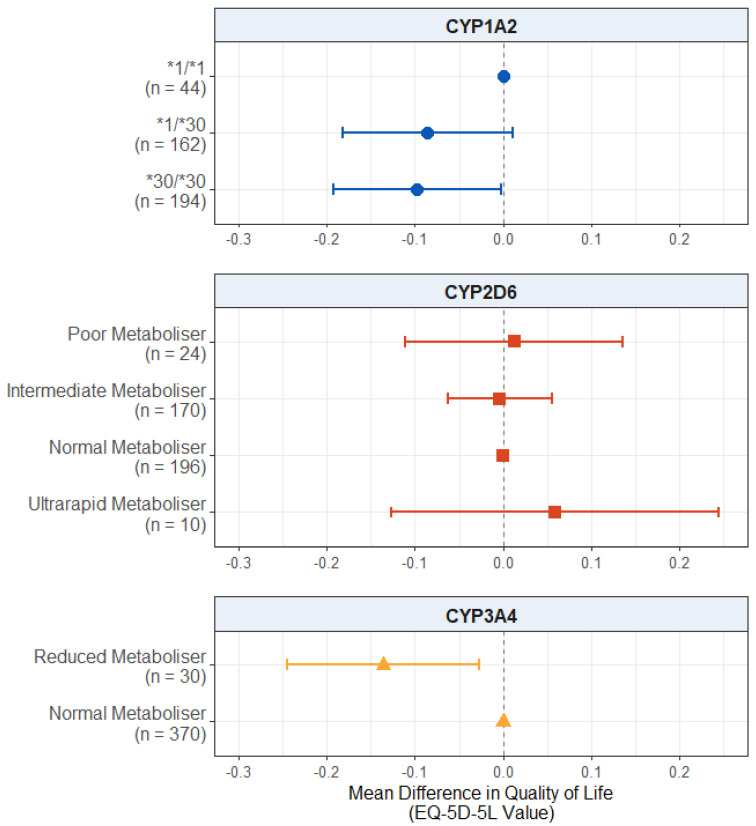
Effect of pharmacogenetic variation in *CYP1A2*, *CYP2D6*, and *CYP3A4* on self-reported health-related quality of life in participants with a psychosis diagnosis. Comparing CYP metaboliser phenotypes/diplotypes on self-reported adverse drug reactions from antipsychotic medications, when accounting for covariates. Reference category is CYP1A2*1/1 diplotype, CYP2D6 normal metaboliser status, and CYP3A4 normal metaboliser status. Number of participants (*n*) in each group is shown in brackets. Abbreviations: *n* = number of participants.

For self-reported quality of life (Figure 5), there was a significant negative effect of the CYP1A2*30/*30 diplotype in this subgroup. Those who carried this diplotype reported lower quality of life scores, on average, compared to the CYP1A2*1/*1 carriers (mean difference: −0.0974; 95% CI: −0.1925, −0.0023; *p* = 0.0447). The effect of the CYP1A2*1/*30 diplotype did not reach significance but passed the suggestive threshold (mean difference: −0.0855; 95% CI: −0.1813, 0.0104; *p* = 0.0807) in the same direction.

In this subgroup, there was also a significant effect of reduced CYP3A4 metabolism, with reduced metabolisers scoring lower on the quality of life scale compared to normal metabolisers (mean difference: −0.1359; 95% CI: −0.2446, −0.0272; *p* = 0.0142).

There was no effect of CYP2D6 metaboliser status (*p* values > 0.532) on the quality of life scores. The full results are presented in Table S22.

#### 2.3.3. Effect of Pharmacogenetic Variation on Medication Dose in Participants with a Diagnosis of Psychosis

**Figure 6:**
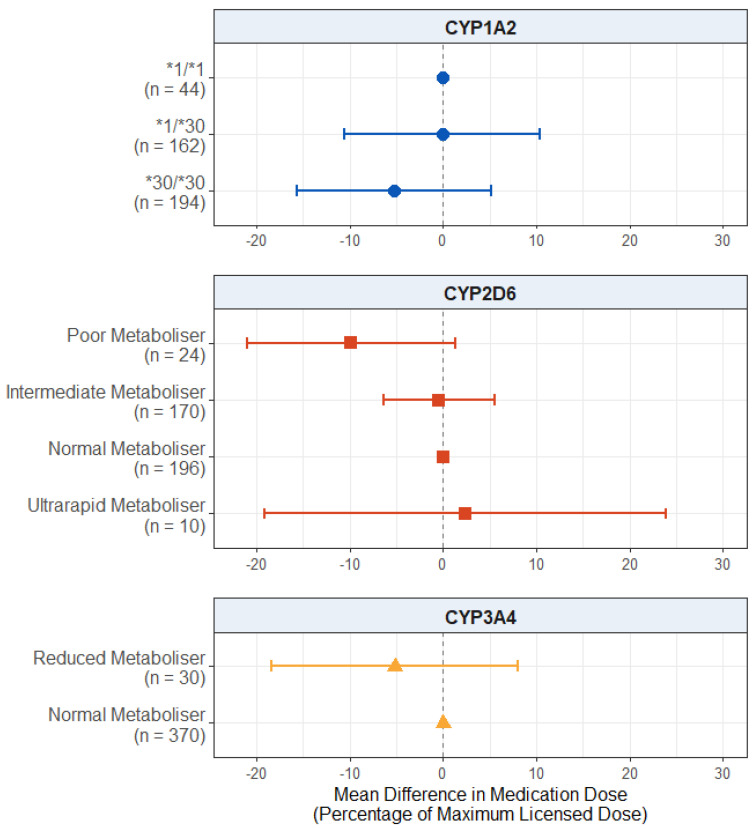
Effect of pharmacogenetic variation in *CYP1A2*, *CYP2D6*, and *CYP3A4* on prescribed antipsychotic medication dose in participants with a psychosis diagnosis. Comparing CYP metaboliser phenotypes/diplotypes on self-reported adverse drug reactions from antipsychotic medications, when accounting for covariates. Reference category is CYP1A2*1/1 diplotype, CYP2D6 normal metaboliser status, and CYP3A4 normal metaboliser status. Number of participants (*n*) in each group is shown in brackets. Abbreviations: *n* = number of participants.

For the antipsychotic medication dose prescribed (Figure 6), we found no significant effect of the *CYP1A2* diplotype (*p* values > 0.321) or reduced CYP3A4 metabolism (*p* = 0.445).

There was also no significant effect of CYP2D6 metaboliser status, but the effect of poor metaboliser status did pass the suggestive evidence threshold (mean difference: −9.854; 95% CI: −21.040, 1.331; *p* = 0.0850), with the poor metabolisers being prescribed lower average antipsychotic doses compared to the normal metabolisers. The full results are presented in Table S23.

## 3. Discussion

The Pharmacogenetics in Mental Health study [29] aims to investigate a pharmacogenetic intervention for people with psychosis and/or taking antipsychotic medications in UK mental health services. In this paper, we present analyses of the baseline data. We hypothesised that an individual’s pharmacogenetic profile could influence antipsychotic treatment outcomes before their genetic make-up was made known to the participants and their clinicians.

### 3.1. Presence of Non-Normal Function Variants

When looking at *CYP2D6* and *CYP3A4* only, most of our participants (63%) carried at least one potentially clinically relevant star allele (Table 3), designated as non-normal function with the highest level of evidence quality by the Pharmacogenomics Knowledgebase (PharmGKB). This was mostly driven by *CYP2D6*, the main pharmacogene for antipsychotic medications, which is also involved in the metabolism of ~20% of commonly used drugs across a broad spectrum of medical disciplines including psychiatry, pain management, oncology, and cardiology [41].

### 3.2. Effect on Adverse Antipsychotic Drug Reactions

Our first hypothesis was that CYP enzyme activity is negatively associated with adverse antipsychotic drug reactions. We found evidence that CYP2D6 ultrarapid metabolisers reported fewer adverse drug reactions compared to normal metabolisers (Figure 1), supporting this hypothesis. CYP2D6 ultrarapid metabolisers process substrates of this enzyme (which include most commonly used antipsychotic medications) at an increased rate relative to normal metabolisers, leading to lower levels of the active drug reaching systemic circulation and faster clearance of these substrates from the body [19]. While this is typically associated with less symptom improvement and decreased efficacy from the medications [16], we could assume that ultrarapid metabolisers are more likely to experience fewer effects from the medications more broadly, including adverse reactions.

Interestingly, when looking at the effect on the individual LUNSERS domains, the effect of CYP2D6 ultrarapid metaboliser status did differ for some domains. There were strong effects on the extrapyramidal, autonomic, psychic, and hormonal domains (in the same direction as the full analysis) but no effect on the anticholinergic, allergic, or miscellaneous domains (Appendix B). This proposes the idea that the effect of CYP2D6 metabolism may be more relevant for certain types of adverse effects than others, though it may also be linked to the side effects of the individual medications themselves. More research is needed focusing on specific sets of adverse effects to clarify this.

Previous evidence that has found an association between CYP2D6 metaboliser status and antipsychotic-induced adverse drug reactions is often seen at the other end of the activity score spectrum, with higher levels of adverse reactions in CYP2D6 poor metabolisers [42]. While we did not find such an association for the full set of adverse drug reactions, there was a trend towards significance for the effect of CYP2D6 poor metaboliser status on the extrapyramidal and anticholinergic domains of the LUNSERS, with the poor metabolisers reporting more of these adverse effects compared to normal metabolisers (Appendix B). There is also evidence from research in pharmacogenetics of antidepressants (another class of medications metabolised by CYP2D6) that CYP2D6 ultrarapid metabolisers may be at an increased risk of suicide compared to normal metabolisers [43], suggesting non-optimal treatment response.

This effect of CYP2D6 ultrarapid metaboliser status remained significant when the sample was restricted to just those with a diagnosis of psychosis (Figure 4). There was also a suggestive effect of CYP3A4 reduced metabolism, with those in this group reporting more adverse drug reactions compared to normal metabolisers. Though not passing the *p* = 0.05 significance threshold, this also supports our hypothesis. While CYP3A4 is not the primary enzyme involved in the metabolism of most antipsychotic medications (with the exception of quetiapine), it is involved to a lesser extent in the metabolism of many antipsychotics [44], as well as up to 50% of all commonly prescribed medications [45]. The higher antipsychotic medications doses prescribed in the individuals with a diagnosis of psychosis compared to those without may explain why this result was only seen in this restricted sample.

### 3.3. Effect on Quality of Life

Our second hypothesis was that extreme metabolisers will report lower quality of life scores. We did not find significant evidence of an effect of pharmacogenetic variation in any of the three genes on the participants’ quality of life in the full sample (Figure 2). There was, however, weak evidence that carrying one or two copies of the inducible CYP1A2*30 allele was associated with lower quality of life scores.

CYP1A2 is involved in the metabolism of only a few antipsychotic medications, notably, clozapine and olanzapine, though no pharmacogenetic guidelines have been provided [15,16]. Over a third of this sample was taking either clozapine or olanzapine (full sample: 35.3%; psychosis only: 38.8%), which may explain this finding. The mechanism by which this diplotype leads to lower self-reported quality of life scores is, however, unclear. The CYP1A2*30 allele is the inducible allele (previously CYP1A2*1F), with higher activity in the presence of a CYP1A2 inducer, such as carbamazepine or tobacco smoking. In cases where someone carries the CYP1A2*30/*30 diplotype and takes an inducer, they would be expected to have increased CYP1A2 enzyme activity. We propose that this may lead to reduced blood levels of medication and less symptom improvement, which may then lead to reduced quality of life scores. Differences in the experiences of adverse reactions not included on the scale used in the present study (LUNSERS) may also be implicated. This explanation is presented cautiously, as *CYP1A2* showed no other significant effects on the other outcomes we measured, and we were unable to measure blood concentrations of medications or symptom changes, two outcomes that could help to elucidate this association. It is likely that this relationship involves a complex drug–drug–gene interaction process due to the inducibility of the allele, with potential influences from both psychiatric and non-psychiatric medications, as well as lifestyle factors (e.g., smoking).

In the psychosis-diagnosis-only sample (Figure 5), there was evidence that CYP3A4 reduced metabolism was associated with lower quality of life scores. Reduced CYP3A4 activity also led to higher scores on the adverse drug reaction scale, which may mediate this relationship between reduced metabolism and lower quality of life scores, though further research is needed to confirm this.

### 3.4. Effect on Medication Dose

Our final hypothesis was that CYP activity is positively associated with the antipsychotic medication dose prescribed. In the subgroup with psychosis, we found suggestive evidence that CYP2D6 poor metabolisers were prescribed lower average antipsychotic doses compared to the normal metabolisers by almost 10 percentage points as a proportion of the maximum licensed dose according to the British National Formulary (BNF) guidelines (Figure 6). While tentative and needing replication, this result is in line with our hypothesis, as well as previous evidence that clinicians may empirically prescribe medications in line with a patient’s metaboliser status even before this is known [46,47,48]. However, we did not find this effect in the overall sample (Figure 3), possibly due to the wider range and lower average doses prescribed to the participants who did not have psychosis.

Where pharmacogenetic recommendations are available for antipsychotic medications, they typically suggest dose reductions for poor metabolisers, with some guidelines available for intermediate metabolisers (dose reductions) and ultrarapid metabolisers (increased dose or choosing an alternative medication) [15,16]. Previous evidence suggests that dose reductions may occur for those with lower enzyme activity through the trial-and-error approach to antipsychotic medication prescribing but to a lesser extent than the guidelines recommend [46,48]. Our findings are also in line with this, though the evidence was weak, suggesting limited evidence of doses aligning with the CYP phenotype over time. Moreover, even if a person’s dose does eventually become adapted to their pharmacogenetic phenotype through usual prescribing, this may require repeated drug trials over a long period of time. Together, this may mean that individuals are treated with non-optimal medications and/or doses for prolonged periods. A pre-emptive pharmacogenetics-guided approach, on the other hand, could identify optimal drugs and doses more promptly.

### 3.5. Strengths

This is, to the best of our knowledge, the first UK-based study of the implementation of pharmacogenetic testing to inform the prescribing of antipsychotic medications. The evidence of clinical benefits of pharmacogenetics in psychosis is limited [20], and we aimed to increase the understanding of how pharmacogenetic variation can influence outcomes for antipsychotic treatment in a UK setting, specifically.

We studied multifaceted outcomes relevant to participants (adverse drug reactions, quality of life, and antipsychotic dose prescribed) at the time of study enrolment. Unlike previous studies, this was a diverse sample, with participants from a wide range of ethnicity groups and ages and both men and women, which aids the generalisability of the findings.

Finally, we also looked at the effect of pharmacogenetic variation across a number of antipsychotic-relevant pharmacogenes and accounted for the effects of phenoconversion on enzyme activity. This was performed to capture a more accurate representation of an individual’s CYP enzyme activity at the time of the assessment (rather than solely based on genetic profile and excluding the impact of known environmental influences).

### 3.6. Limitations

Firstly, we only included strong and moderate inhibitors/inducers when adjusting for phenoconversion, meaning that there may have been some small effects of weak inducers/inhibitors that were not accounted for. There are also other factors that can result in altered enzyme activity for each of the CYP enzymes analysed, such as liver functioning/disease [49], dietary factors, or cancer [50], which we were unable to account for.

Another limitation is that we did not measure symptom changes directly. This is another key treatment outcome, and previous evidence has suggested that CYP2D6 metaboliser status may be associated with symptom improvement after antipsychotic treatment in adolescents [51]. We were also unable to determine whether there were any differences in blood concentrations of medications between the different metaboliser status groups, as therapeutic drug monitoring is not routinely carried out in the UK for antipsychotics apart from for clozapine, though experts have made proposals for its broader use in other countries [52,53]. Plasma concentrations have been found to differ between the pharmacogenetic profiles and may provide an explanation for the clinical features we were able to analyse [54] by providing more insight into the pharmacokinetics of the medications [55] and the effect of metabolism status on this.

In our analyses, we controlled for the antipsychotic medication taken and the dose prescribed, but due to sample size limitations (and a low percentage of extreme metabolisers), we had to analyse all the antipsychotics as a group and were unable to study the effect of pharmacogenetic variation on specific antipsychotic drugs. It is possible that differences in the effects of pharmacogenetic variation on each of the outcomes were present between the different medications taken in the sample (particularly between those primarily metabolised by different CYP enzymes). Due to the considerable number of different medications prescribed (including approximately 18% of participants being prescribed two different antipsychotic medications), we were unable to carry out stratified analyses by medication. Effects of the different antidepressant and mood stabiliser agents used in the sample may also have contributed to effects that we were also unable to account for (with the exception of the phenoconversion effects from inhibitors/inducers of the enzymes of interest, such as fluoxetine).

While the sample used was diverse in ethnicity, reflecting the distribution of ethnic groups seen in the general population of England and Wales [56], the majority of the sample were still of White ethnicity. This means that we cannot be fully sure that the results found equally reflect the associations seen in each of the ethnicity groups present in the sample, and we were unable to look at the effects in each ethnic group individually.

### 3.7. Clinical Implications

Pharmacogenetic recommendations for clinical practice are still in development, with some already provided by the Dutch Pharmacogenetics Working Group (DPWG) [16] and the Food and Drug Administration (FDA) [15]. The Clinical Pharmacogenetics Implementation Consortium (CPIC) guideline for antipsychotics is also currently still in progress (as of June 2025) [57]. Alongside the pressing need for the discovery of new medications for mental health [58,59,60], pharmacogenetics could help optimise the use of medications already available for a more tailored treatment approach to the individual patient.

### 3.8. Future Directions

This study was an initial investigation into whether there is an association between pharmacogenetic variation and treatment outcomes at baseline, before the pharmacogenetic test results have been delivered to an individual’s clinician. Follow-up data from after implementation of the pharmacogenetic report also needs to be analysed to investigate whether this can improve treatment outcomes. Positive results have been found for the use of pharmacogenomic testing in antidepressant treatment, particularly for those with more severe symptoms or a history of poor treatment response, but currently there is no available evidence in antipsychotic treatment or pharmacogenetic implementation within mental health services in the UK [61].

Future studies should also include measures of treatment outcomes beyond those included in the present study. Measuring plasma concentrations of medication in participants’ blood and treatment efficacy using psychosis symptom scales would allow for the mechanisms by which an individual’s pharmacogenetic profile impacts their response to medication to be investigated in further detail.

Where sample sizes allow, stratified and/or interaction analyses should be considered to determine if there are any differences in the effect of pharmacogenetic variation on antipsychotic treatment outcomes between different antipsychotic agents, between different ethnicity/ancestry groups, and between those taking one medication compared to those taking multiple.

## 4. Materials and Methods

### 4.1. Participants

The sample for this study was taken from the Pharmacogenetics: Genetics and Environment in Mental Health Study [29]. This is an ongoing prospective cohort study to investigate the effect of a pharmacogenetic intervention on antipsychotic prescribing and treatment outcomes. The participants were recruited from National Health Service clinics across England and were introduced to the study either through clinician referral or self-referral between November 2021 and February 2025. Participants were eligible if they were (1) over the age of 18; (2) diagnosed with a psychotic disorder (schizophrenia, schizoaffective disorder, bipolar disorder with history of psychosis, or other psychoses) and/or taking an antipsychotic medication and/or due to start an antipsychotic medication; and (3) had the capacity to consent. Participants could be taking any antipsychotic medication, at any dose, and no adjustments were made to their medication at the time of recruitment.

The present study used data collected from baseline assessments only (i.e., before a report with pharmacogenetic test results was delivered to their clinician). The sample was restricted to only those taking antipsychotic medications at baseline. All the participants provided a DNA sample, either as a blood or saliva sample.

### 4.2. Ethical Considerations

All the participants signed an informed consent form at the baseline assessment. Ethical approval was given for this study by the London–Camden and Kings Cross Research Ethics Committee on 28th October 2019 (reference number: 19/LO/1403).

### 4.3. Treatment Outcomes

We measured three antipsychotic treatment outcomes: (1) adverse drug reactions to antipsychotic medications; (2) quality of life; and (3) medication dose prescribed. Each is briefly outlined below, and more information is provided in Appendix A.

#### 4.3.1. Adverse Antipsychotic Effects

The participants completed the Liverpool University Neuroleptic Side Effects Rating Scale (LUNSERS), a 51-item self-administered scale comprised of 41 common antipsychotic adverse drug reactions and 10 “red herring” items (Table S24) [36]. The outcome used in the present study was a scaled total score of the 41 side effect items. This was calculated as the total each participant scored as a proportion of the maximum they could have scored, based on the number of items they answered. The scale is split into seven domains (extrapyramidal, anticholinergic, other autonomic, allergic reactions, psychic, hormonal, and miscellaneous), which were also analysed individually to determine if there were any differences in the effects of pharmacogenetic variation on different subsets of adverse antipsychotic drug reactions.

#### 4.3.2. Quality of Life

To measure quality of life, the participants completed the five-level version of the EuroQol scale (EQ-5D-5L), a five-item self-report scale that measures aspects of the individual’s health on that day [37,62]. Scores across the five items can be consolidated into a single numerical value, based on the weighted relative importance of impairment on each domain. These weightings are available in value sets for various countries; the most recent English value set was used to generate the scores [38,39], which were used as the outcome.

#### 4.3.3. Medication Dose

The participants reported their antipsychotic prescription(s), and the doses were converted into a percentage of the British National Formulary (BNF) maximum licensed dose (Table S25) [40]. The BNF is the main reference tool used by prescribers in the UK, providing information to aid in the selection, prescription, and administration of medications, including the maximum licensed dose. For individuals taking multiple antipsychotic medications, the percentages of each were summed to create a total percentage score. Any medications prescribed “when required” were not included.

### 4.4. Genotyping

#### 4.4.1. DNA Extraction and Quality Verification

DNA extraction and quality control were carried out at University College London Genomics (London, UK). DNA was extracted from thawed whole blood (250 μL) or saliva (1 mL) using an automated HID NIMBUS^®^ Presto^®^ System (Hamilton, Reno, NV, USA; Thermo Fisher Scientific, Waltham, MA, USA) using the Mag-Bind^®^ Blood & Tissue DNA HDQ 96 Kit (Omega Bio-tek, Norcross, GA, USA). Extracted nucleic acid was eluted in 54 μL or 100 μL of elution buffer for blood and saliva, respectively. The samples were extracted in batches of 96 for blood samples and 24 for saliva samples.

Following DNA extraction, the eluted DNA samples underwent quality control to assess the concentration on the Qubit platform (Inivitrogen™, Thermo Fisher Scientific, Waltham, MA, USA), using the Broad Range DNA protocol, using 2 μL of the eluted material. DNA integrity was assessed using the Genomic DNA ScreenTape System, again using 2 μL of the eluted material. The samples were processed if they had concentrations above 30 ng/μL and DNA integrity numbers higher than 6.

#### 4.4.2. Sample Preparation and Pharmacogenetic Analysis

For the pharmacogenetic data (i.e., star alleles and metaboliser status), the results were obtained from two separate laboratories, both of which used the Agena MassARRAY^®^ genotyping platform. The main laboratory used (82% of samples) utilised the VeriDose^®^ Core Panel and the VeriDose^®^ CYP2D6 Copy Number Variant (CNV) Panel (Agena Bioscience, USA) to characterise the genes of interest. The other laboratory used a modified panel, which cannot be disclosed due to commercial licensing.

### 4.5. Pharmacogenetic Classification

We used the genotype and phenotype data to categorise the participants into the pharmacogenetic groups. As enzyme function can be inhibited (decreased) and/or induced (increased) by external factors, such as concomitant medication and tobacco smoking (phenoconversion), this was accounted for, where possible, to give a more accurate measure of enzyme activity [63,64]. Inducers/inhibitors were taken from the Drug Interactions Flockhart Table (https://drug-interactions.medicine.iu.edu/MainTable.aspx (accessed on 5 June 2025)). Smoking status was ascertained using participant self-report or medical records to determine whether the participants were smoking anything containing tobacco at the time of the assessment or if they had given up smoking in the previous month (due to potential lasting effects). Participants who had given up smoking more than a month before or who reported smoking something not containing tobacco were classified as non-smokers.

For *CYP1A2*, we used the new categorisation of star alleles, published by the Pharmacogene Variation Consortium (PharmVar) in December 2024 [30,31]. No clinical functions have been assigned to the new star alleles, so we used the diplotypes identified to categorise the groups based on the number of copies of the CYP1A2*30 allele carried by the participants. This is an inducible allele (formerly CYP1A2*1F), meaning increased function is expected in the presence of a CYP1A2 inducer, such as carbamazepine or tobacco smoking. To account for phenoconversion, separate variables were created to determine whether an individual took an inhibitor or an inducer of the enzyme to be included in the model as covariates.

For CYP2D6, we used the star alleles to generate a CYP2D6 activity score per participant [65]. This was adjusted directly for phenoconversion (reduced by 80% for those taking a strong inhibitor and 50% for those taking a moderate inhibitor) and reclassified into the metaboliser status groups. The translations from activity score into metaboliser status were provided by CPIC and PharmGKB [66].

For CYP3A4, individuals were classified as intermediate metabolisers if they carried one copy of the CYP3A4*22 allele (reduced function) and poor metabolisers if they carried two copies. As only one poor metaboliser was identified in our sample, intermediate and poor metabolisers were combined into a “reduced metabolism” group for analysis. Separate variables were created to determine whether an individual took an inhibitor or an inducer of CYP3A4.

### 4.6. Statistical Analysis

Analyses were carried out using R (v.4.3.2) [67] to determine the effect of the genes of interest for each outcome measure. Linear regression with robust standard errors was used for the analyses of adverse drug reactions and medication dose due to non-normal data distributions. For the quality of life analyses, a Tobit model was used, as a score of 1 (the highest possible score on the scale) is a censored limit (i.e., not all individuals scoring 1 will have the same level of quality of life, but the scale does not allow for differentiation past this point).

For CYP2D6 and CYP3A4, the metaboliser status was used as a categorical exposure variable, with normal metaboliser as the reference. For *CYP1A2*, the diplotypes were used as the exposure variable, with CYP1A2*1/*1 as the reference. The three pharmacogenes were modelled simultaneously for each outcome to account for their combined effects and to minimise the number of tests carried out. As the three outcomes were assumed to be related (not independent) and the analyses were hypothesis-driven, no correction for multiple testing was applied.

For all the analyses, age, sex, ethnicity, primary mental health diagnosis, antipsychotic medication, concomitant antidepressant prescription dose, concomitant mood stabiliser prescription dose, and concomitant use of CYP1A2 and CYP3A4 inhibitors and/or inducers were included as covariates. For the analyses of adverse drug reactions and quality of life, the total antipsychotic prescription dose was also included. These variables were included to control for the potential effect that they could have on the outcome(s) and/or CYP enzyme activity. More detail is provided in Appendix C.

We also carried out a sensitivity analysis, excluding participants who did not have a diagnosis of a psychotic disorder, to determine if there was any difference in the effect of pharmacogenetic variation on the outcomes in this more homogenous group. The covariates remained the same as the main analysis.

## 5. Conclusions

We have found supporting evidence that the CYP genotype can influence treatment outcomes in those taking antipsychotic medications, even before a pharmacogenetic test has been used. These findings support further research into pharmacogenetic testing for tailoring antipsychotic prescriptions to avoid delays in optimal treatment and make best use of the antipsychotic medications currently available.

## Figures and Tables

**Table 1:** Demographic characteristics of the pharmacogenetics in mental health study sample.

		**All Participants (N = 453)**
Age, mean years (SD)		43.5 (14.5)
	Age range, years	18–82
Sex, *n* (%)		
	Male	245 (54.1%)
	Female	208 (45.9%)
Ethnicity, *n* (%)		
	Asian or Asian British	67 (14.8%)
	Black, Black British, Caribbean, or African	44 (9.7%)
	White	302 (66.7%)
	Mixed or multiple ethnic groups	21 (4.6%)
	Other ethnic group	19 (4.2%)
Primary diagnosis, *n* (%)		
	Schizophrenia	164 (36.2%)
	Bipolar disorder	110 (24.3%)
	Other psychotic disorder	126 (27.8%)
	Other psychiatric disorder	53 (11.7%)
Duration of illness ^1^, mean years (SD)		11.6 (11.1)
	Duration of illness range, years	0–50
Location recruited, *n* (%)		
	Community	277 (61.1%)
	Inpatient	145 (32.0%)
	*Missing*	*31 (6.8%)*
Antipsychotic(s) taken ^2^, *n* (%)		
	Aripiprazole	128 (28.3%)
	Olanzapine	89 (19.6%)
	Quetiapine	82 (18.1%)
	Clozapine	73 (16.1%)
	Risperidone	37 (8.2%)
	Zuclopenthixol	31 (6.8%)
	Amisulpride	23 (5.2%)
	Paliperidone	20 (4.4%)
	Lurasidone	19 (4.2%)
	Flupentixol	17 (3.8%)
	Haloperidol	12 (2.6%)
	Cariprazine	2 (0.4%)
Taking antidepressant(s) ^3^, *n* (%)		190 (42.0%)
Taking mood stabiliser(s) ^3^, *n* (%)		132 (29.1%)

^1^ Duration of illness calculated as the difference between the participant’s age at the time of the baseline assessment and the age they were diagnosed with their classified primary diagnosis. Data missing for 83 (18%) participants. ^2^ In the case that an individual was taking multiple antipsychotics, all have been counted here. ^3^ Further details on the antidepressant medications and mood stabiliser medications taken by the participants are presented in Table S1. Abbreviations: SD = standard deviation; *N/n* = number of participants.

**Table 2:** Cytochrome P450 (CYP) enzyme metaboliser status frequency.

Enzyme/Gene	Metaboliser Status/Diplotype	*n* (%)
CYP2D6	Poor Metaboliser	26 (5.7%)
	Intermediate Metaboliser	198 (43.7%)
	Normal Metaboliser	218 (48.1%)
	Ultrarapid Metaboliser	11 (2.4%)
*CYP1A2*	*1/*1	47 (10.4%)
	*1/*30	183 (40.4%)
	*30/*30	223 (49.2%)
CYP3A4	Poor Metaboliser	1 (0.2%)
	Intermediate Metaboliser	38 (8.4%)
	Normal Metaboliser	414 (91.4%)

Note: For CYP2D6, metaboliser statuses are classified after phenoconversion, where this is possible to account for. Pre-phenoconversion metaboliser status frequencies are available in Table S1. For CYP1A2, the new nomenclature (as of December 2024) has been used to categorise the star alleles, but no metaboliser status groups are available for these, so the groups have been categorised by diplotype only. CYP1A2*30 is the inducible allele.

**Table 3:** Proportion of participants carrying one or more PharmGKB Level 1A non-normal function alleles.

Gene	*n* (%)
*CYP2D6* only	246 (54.3%)
*CYP3A4* only	19 (4.2%)
Both *CYP2D6* and *CYP3A4*	20 (4.4%)
Neither	168 (37.1%)

*CYP2D6* PharmGKB Level 1A non-normal function alleles (DPWG function assignment): *1 × *N* (increased function), *3 (no function), *4 (no function), *5 (no function), *6 (no function), *10 (decreased function), *14 (decreased function), *17 (decreased function), *41 (decreased function). *CYP3A4* PharmGKB Level 1A non-normal function alleles (DPWG function assignment): *20 (no function), *22 (decreased function). Abbreviations: PharmGKB = Pharmacogenomics Knowledgebase.

## Data Availability

The datasets presented in this article are not readily available because the data are part of an ongoing study. Requests to access the datasets should be directed to L.V. (lauren.varney.21@ucl.ac.uk).

## Supplementary Materials

The following supporting information can be downloaded at https://www.mdpi.com/article/10.3390/ph18060892/s1: Table S1. Antidepressant and mood stabilier medications taken in the whole sample and psychosis-diagnosis-only subgroup; Table S2. Cytochrome P450 2D6 (CYP2D6) enzyme metaboliser status frequency before and after phenoconversion in the pharmacogenetics in mental health study sample; Table S3. Star alleles in the panel by Agena Bioscience; Table S4. Cytochrome P450 (CYP) enzyme metaboliser status frequency by ethnic group; Table S5. CYP1A2 nomenclature translations and diplotypes identified per ethnic group; Table S6. CYP3A4 diplotypes identified per ethnic group and associated metaboliser status; Table S7. CYP2D6 diplotpes identified per ethnic group and associated metaboliser status; Table S8. Prevalence of each PharmGKB Level 1A non-normal function variant; Table S9. Effect of pharmacogenetic variation in CYP1A2, CYP2D6, and CYP3A4 on self-reported adverse drug reactions to antipsychotic medications; Table S10. Effect of pharmacogenetic variation in CYP1A2, CYP2D6, and CYP3A4 on self-reported extra pyramidal adverse reactions to antipsychotic medications (LUNSERS Extrapyramidal Domain); Table S11. Effect of pharmacogenetic variation in CYP1A2, CYP2D6, and CYP3A4 on self-reported anticholinergic adverse reactions to antipsychotic medications (LUNSERS Anticholinergic Domain); Table S12. Effect of pharmacogenetic variation in CYP1A2, CYP2D6, and CYP3A4 on self-reported other autonomic adverse reactions to antipsychotic medications (LUNSERS Other Autonomic Domain); Table S13. Effect of pharmacogenetic variation in CYP1A2, CYP2D6, and CYP3A4 on self-reported allergic adverse reactions to antipsychotic medications (LUNSERS Allergic Reactions Domain); Table S14. Effect of pharmacogenetic variation in CYP1A2, CYP2D6, and CYP3A4 on self-reported psychic adverse reactions to antipsychotic medications (LUNSERS Psychic Domain); Table S15. Effect of pharmacogenetic variation in CYP1A2, CYP2D6, and CYP3A4 on self-reported hormonal adverse reactions to antipsychotic medications (LUNSERS Hormonal Domain); Table S16. Effect of pharmacogenetic variation in CYP1A2, CYP2D6, and CYP3A4 on self-reported miscellaneous adverse reactions to antipsychotic medications (LUNSERS Miscellaneous Domain); Table S17. Effect of pharmacogenetic variation in CYP1A2, CYP2D6, and CYP3A4 on self-reported health-related quality of life; Table S18. Effect of pharmacogenetic variation in CYP1A2, CYP2D6, and CYP3A4 on prescribed antipsychotic medication dose; Table S19. Demographic characteristics of the psychosis diagnosis only subgroup of the pharmacogenetics in mental health study; Table S20. Cytochrome P450 (CYP) enzyme metaboliser status frequency in the psychosis diagnosis only subgroup; Table S21. Effect of pharmacogenetic variation in CYP1A2, CYP2D6, and CYP3A4 on self-reported adverse drug reactions to antipsychotic medications in participants with a diagnosis of psychosis only; Table S22. Effect of pharmacogenetic variation in CYP1A2, CYP2D6, and CYP3A4 on self-reported health-related quality of life in participants with a diagnosis of psychosis only; Table S23. Effect of pharmacogenetic variation in CYP1A2, CYP2D6, and CYP3A4 on prescribed antipsychotic medication dose in participants with a diagnosis of psychosis only; Table S24. Liverpool University Neuroleptics Side Effects Rating Scale (LUNSERS) Items; Table S25. Maximum British National Formulary (BNF) doses of antipsychotic medications; Table S26. Maximum British National Formulary (BNF) doses of antidepressant medications; Table S27. Maximum British National Formulary (BNF) doses of mood stabiliser medications; Table S28. Descriptive statistics for all covariates used in regression models.

## Appendix A

Adverse Effects—Liverpool University Neuroleptics Side Effects Rating Scale

The Liverpool University Neuroleptics Side Effects Rating Scale (LUNSERS) [36] consists of 51 items to measure adverse drug reactions to antipsychotic medications. The scale consists of 41 common side effects to antipsychotic medications and 10 “red herring” items. The 41 side effects are separated into seven subcategories: extrapyramidal side effects; anticholinergic side effects; other autonomic side effects; allergic reactions; psychic side effects; hormonal side effects; and miscellaneous side effects (side effects that fit into each subcategory and the red herrings are presented in Table S24).

Each item is scored on a five-point scale from “Not at all” (scored as 0) to “Very much” (scored as 4), based on how much the individual has experienced that side effect in the previous month. Due to there being two items related to menstruation that are only relevant for ~50% of the sample, and missing data for some side effects that the participants commonly declined to answer, the main outcome was a scaled score for the participants, rather than the raw total score. This was calculated by taking the value participants scored as a percentage of the maximum they could have scored, based on the number of items that they answered. Participants who did not answer more than 10 items (*n* = 2) were removed from the analysis.

For example, if a non-menstruating participant declined to answer three questions and scored 55 across the responses that they did give, their maximum possible score for the items that they did give would be 164 − (4 × 5) = 144, and so their scaled score would be 55/144 = 38.2. In comparison, a menstruating participant who answered all the questions and also scored 55 would have a scaled score of 55/164 = 33.5.

Quality of Life—EQ-5D-5L

The EQ-5D-5L is a measure of self-reported health status or health-related quality of life [37,62]. It consists of two parts: a five-item questionnaire related to general aspects of a person’s functioning (namely, mobility, self-care, usual activities, pain/discomfort, and anxiety/depression), which can be converted into either EQ-5D profiles or EQ-5D values; and a visual analogue scale (EQ VAS) that captures the individual’s overall assessment of their health on that day on a scale from 0 (worst possible) to 100 (best possible).

For this study, the EQ-5D values were used as the outcome. This requires the responses given across each of the five dimensions (the EQ-5D profile) to be converted into a single value (the EQ-5D value), based on the relative importance a population is assumed to place on impairment in each category (the value set). These value sets are available for multiple countries; for this analysis, the most recent EQ-5D-5L English value set was used. Scores can range from 1 (no impairment) to −0.285 (highest level of impairment on all five dimensions). Further explanation of how these value sets are determined and applied is available in Devlin et al. (2018) [38].

Medication Dose

The medication dose outcome was calculated as the participants’ reported antipsychotic prescription dose as a percentage of the maximum licensed dose as per the British National Formulary (BNF) [40]. When an individual was prescribed more than one antipsychotic medication, the percentages for each medication were summed together. Only regularly taken medications were included (i.e., any medications noted as PRN/when required were not included in the percentages, as detail was lacking on how often these medications were taken). The BNF maximum licensed doses used are provided in Table S25, based on the latest available guidelines (as of December 2024).

For example, if an individual was taking 10 mg of olanzapine every day, their BNF percentage would be 50%. If the same person was also taking 15 mg of aripiprazole each day (50%), their total BNF percentage would be 100%. This approach was also used to combine depot medications and tablets (e.g., a 200 mg depot of zuclopenthixol per week (33%) combined with 10 mg tablets of aripiprazole per day (33%) would be 67% of the maximum).

The same process was also used to quantify antidepressant BNF percentages and mood stabiliser BNF percentages, which were each used as covariates in all the analyses. The BNF maximum licensed doses used to calculate these are presented in Table S26 (antidepressants) and Table S27 (mood stabilisers).

## Appendix B

When looking at the effect of pharmacogenetic variation on adverse antipsychotic drug reactions within each of the Liverpool University Neuroleptics Side Effects Rating Scale (LUNSERS) domains (extrapyramidal, anticholinergic, other autonomic, allergic, psychic, hormonal, and miscellaneous; Table S24), the general pattern of effect remained consistent across the majority of the domains. However, there were some differences worth noting. In particular, for some domains there was a trend towards increased adverse reaction reporting by CYP2D6 poor metabolisers, which is also in line with our hypothesis of a negative association between CYP activity and adverse antipsychotic drug reactions. The effect of CYP2D6 ultrarapid metaboliser status and fewer reported adverse effects also was not seen across all the domains, with no effect of CYP2D6 ultrarapid metaboliser status seen for the anticholinergic, allergic, or miscellaneous domains.

Extrapyramidal Effects

For the effect on extrapyramidal effects (e.g., muscle stiffness, restlessness, shakiness), there was significant evidence that CYP2D6 ultrarapid metabolisers reported fewer extrapyramidal effects compared to normal metabolisers (mean difference: −12.9; 95% CI: −21.9, −4.0, *p* = 0.00484). There was also a trend towards a positive effect of CYP2D6 poor metaboliser status on reported extrapyramidal effects (mean difference: 6.7; 95% CI: −0.9, 14.5, *p* = 0.0864). There was no effect of CYP2D6 intermediate metaboliser status (*p* = 0.917).

There was no evidence of an effect of CYP1A2 diplotype (*p* values > 0.160) or reduced CYP3A4 metabolism (*p* = 0.169). The full results are presented in Table S10.

Anticholinergic Effects

For anticholinergic effects (e.g., dry mouth, blurred vision, passing a lot of water), we found suggestive evidence that CYP2D6 poor metabolisers reported more anticholinergic effects compared to normal metabolisers (mean difference: 6.7; 95% CI: −0.8, 14.2, *p* = 0.0808). There was no evidence of an effect of CYP2D6 ultrarapid metaboliser status (*p* = 0.491) or intermediate metaboliser status (*p* = 0.490).

There was no evidence of an effect of CYP1A2 diplotype (*p* values > 0.116) or reduced CYP3A4 metabolism (*p* = 0.248). The full results are presented in Table S11.

Other Autonomic Effects

We found significant evidence that CYP2D6 ultrarapid metabolisers reported fewer other autonomic effects on the LUNSERS (e.g., dizziness, palpitations, increased sweating) compared to normal metabolisers (mean difference: −15.5; 95% CI: −22.5, −8.4, *p* = 0.0000223). There was no evidence of an effect of CYP2D6 poor metaboliser status (*p* = 0.433) or intermediate metaboliser status (*p* = 0.994).

There was no evidence of an effect of CYP1A2 diplotype (*p* values > 0.540) or reduced CYP3A4 metabolism (*p* = 0.428). The full results are presented in Table S12.

Allergic Reactions

For the allergic reactions domain (e.g., rash, sensitivity to the sun, itchy skin), we found no evidence of CYP2D6 metaboliser status (*p* values > 0.439), CYP1A2 diplotype (*p* values > 0.747), or reduced CYP3A4 metabolism (*p* = 0.260). The full results are presented in Table S13.

Psychic Effects

For the psychic effects (e.g., tension, sleeping too much, difficulty remembering things), we found that CYP2D6 ultrarapid metabolisers reported significantly less of these effects compared to normal metabolisers (mean difference: −15.6; 95% CI: −28.8, −2.4, *p* = 0.0214). There was no evidence of an effect of CYP2D6 poor metaboliser status (*p* = 0.267) or intermediate metaboliser status (*p* = 0.556).

There was no evidence of an effect of CYP1A2 diplotype (*p* values > 0.265) or reduced CYP3A4 metabolism (*p* = 0.295). The full results are presented in Table S14.

Hormonal Effects

We found significant evidence that CYP2D6 ultrarapid metabolisers reported fewer hormonal effects (e.g., period problems, increased sex drive, swollen or tender chest) compared to normal metabolisers (mean difference: −10.1; 95% CI: −18.1, −2.1, *p* = 0.0136). There was no evidence of an effect of CYP2D6 poor metaboliser status (*p* = 0.107) or intermediate metaboliser status (*p* = 0.143).

There was no evidence of an effect of CYP1A2 diplotype (*p* values > 0.441) or reduced CYP3A4 metabolism (*p* = 0.216). The full results are presented in Table S15.

Miscellaneous Effects

For the miscellaneous effects domain (e.g., headaches, losing weight, putting on weight), we found no evidence of CYP2D6 metaboliser status (*p* values > 0.134), CYP1A2 diplotype (*p* values > 0.451), or reduced CYP3A4 metabolism (*p* = 0.736). The full results are presented in Table S16.

## Appendix C

Age, sex, ethnicity, primary mental health diagnosis, antipsychotic medication, concomitant antidepressant prescription dose, concomitant mood stabiliser prescription dose, and concomitant use of any inhibitors and/or inducers of the genes were included as covariates in all the analyses. For the analyses of adverse drug reactions and quality of life, the total antipsychotic prescription dose was also included as a covariate. Descriptive statistics of all the covariates are presented in Table S28.

All the medication dose variables (antidepressant, mood stabiliser, and antipsychotic doses) were included as continuous variables. Age was included as a continuous variable; sex was included as a binary variable (male/female). CYP1A2 inducer, CYP3A4 inhibitor, and CYP3A4 inducer variables were included in the model as binary variables (yes/no). No participants in the sample were taking a strong or moderate CYP1A2 inhibitor, so this variable was dropped. CYP2D6 inhibitors were accounted for in the generation of the phenoconverted metaboliser status groups, so a separate variable was not included in the models. Ethnicity was included as a categorical variable, with the following levels: (1) Asian or Asian British; (2) Black, Black British, Caribbean, or African; (3) White; (4) Mixed or multiple ethnic groups; and (5) Other ethnic group. Primary mental health diagnosis was included as a categorical variable, with the following levels: (1) schizophrenia; (2) bipolar disorder; (3) other psychotic disorder (e.g., schizoaffective disorder, depression with psychotic features); and (4) other psychiatric disorder (e.g., obsessive–compulsive disorder, emotionally unstable personality disorder). Antipsychotic medication was included as a categorical variable, with the following levels: (1) aripiprazole; (2) clozapine; (3) olanzapine; (4) quetiapine; (5) other antipsychotic (e.g., risperidone, lurasidone, zuclopenthixol); and (6) multiple antipsychotics (i.e., all individuals regularly prescribed two or more different antipsychotic medications).
