# Supplementary material for: Effect of CYP1A2, CYP2D6, and CYP3A4 Variation on Antipsychotic Treatment Outcomes
Source: Pharmaceuticals (Basel). 2025 Jun 14;18(6):892. doi: 10.3390/ph18060892 (PMC12196257; doi:10.3390/ph18060892)
Supplement: Supplementary file 1 [file pharmaceuticals-18-00892-s001.zip › pharmaceuticals-3656369-supplementary.pdf]

Table S1. Antidepressant and Mood Stabilier Medications Taken in the Whole Sample and Psychosis Diagnosis-Only Subgroup

	Full Sample	Psychosis Diagnosis-Only Subgroup
**Antidepressant medication[1], n (%)**	190 (42.0%)	147 (36.8%)
Agomelatine	1 (0.2%)	-
Amitriptyline	9 (2.0%)	8 (2.0%)
Citalopram	15 (3.3%)	12 (3.0%)
Clomipramine	1 (0.2%)	-
Dosulepin	1 (0.2%)	1 (0.2%)
Duloxetine	13 (2.9%)	11 (2.8%)
Escitalopram	9 (2.0%)	7 (1.8%)
Fluoxetine	23 (5.1%)	20 (5.0%)
Imipramine	1 (0.2%)	1 (0.2%)
Lofepramine	1 (0.2%)	1 (0.2%)
Mirtazapine	38 (8.4%)	29 (7.2%)
Paroxetine	3 (0.7%)	3 (0.8%)
Sertraline	47 (10.4%)	36 (9.0%)
Trazodone	2 (0.4%)	2 (0.5%)
Venlafaxine	34 (7.5%)	24 (6.0%)
Vortioxetine	14 (3.1%)	8 (2.0%)
**Mood stabiliser medication[1], n (%)**	132 (29.1%)	125 (31.3%)
Carbamazepine	9 (2.0%)	8 (2.0%)
Lamotrigine	30 (6.6%)	29 (7.2%)
Lithium carbonate	55 (12.1%)	50 (12.5%)
Sodium valproate	53 (11.7%)	53 (13.2%)

[1] In the case that an individual is taking multiple antidepressants/mood stabilisers, all have been counted here.

Table S2. Cytochrome P450 2D6 (CYP2D6) Enzyme Metaboliser Status Frequency Before and After Phenoconversion in the Pharmacogenetics in Mental Health Study Sample

**Before Phenoconversion Adjustment, *n* (%)**		**After Phenoconversion Adjustment, *n* (%)**	
Poor Metaboliser	26 (5.7%)	Poor Metaboliser	26 (5.7%)
Intermediate Metaboliser	176 (38.9%)	Intermediate Metaboliser	198 (43.7%)
Normal Metaboliser	238 (52.5%)	Normal Metaboliser	218 (48.1%)
Ultrarapid Metaboliser	13 (2.9%)	Ultrarapid Metaboliser	11 (2.4%)

Table S3. Star Alleles on the Panel by Agena Bioscience

Gene	Alleles
**CYP1A2**[1]	*Old nomenclature*: *1C, *1F, *1K, *1L, *7, *11<br>*New nomenclature*: *30
**CYP2D6**	*2, *3, *4, *5, *6, *7, *8, *9, *10, *11, *12, *14, *15, *17, *18, *19, *20, *29, *36, *41, *69, *114
**CYP3A4**	*2, *17, *22

Note: Alleles on the unmodified Agena MassARRAY panel, which was used to genotype 82% of the sample

[1] The nomenclature for the CYP1A2 star alleles was changed as of December 2024, but most participants were genotyped before this change, meaning they were genotyped on a panel using the old nomenclature. Alleles on the panels under both the old and the new nomenclature have been presented. More information on how the star alleles were translated into the new nomenclature is available in Table S4.

Table S4. Cytochrome P450 (CYP) Enzyme Metaboliser Status Frequency By Ethnicity Group

Gene	Metaboliser Status	Ethnicity Group, *n* (%)				
		Asian	Black	White	Mixed or Multiple	Other
**CYP2D6**	Poor Metaboliser	1 (1.5%)	1 (2.3%)	24 (7.9%)	0	0
	Intermediate Metaboliser	18 (26.9%)	18 (40.9%)	144 (47.7%)	11 (52.4%)	7 (36.8%)
	Normal Metaboliser	47 (70.1%)	20 (45.5%)	130 (43.0%)	10 (47.6%)	11 (57.9%)
	Ultrarapid Metaboliser	1 (1.5%)	5 (11.4%)	4 (1.3%)	0	1 (5.3%)
**CYP1A2**	*1/*1	14 (20.9%)	4 (9.1%)	23 (7.6%)	3 (14.3%)	3 (15.8%)
	*1/*30	25 (37.3%)	26 (59.1%)	113 (37.4%)	8 (38.1%)	11 (57.9%)
	*30/*30	28 (41.8%)	14 (31.8%)	166 (55.0%)	10 (47.6%)	5 (26.3%)
**CYP3A4**	Poor Metaboliser	0	0	1 (0.3%)	0	0
	Intermediate Metaboliser	0	0	36 (11.9%)	0	2 (10.5%)
	Normal Metaboliser	67 (100%)	44 (100%)	265 (87.7%)	21 (100%)	17 (89.5%)

Note: For CYP2D6, metaboliser statuses are classified after phenoconversion, where this is possible to account for. Genotype-based metaboliser statuses (before phenoconversion) are presented in Table S1. Percentages are given per each ethnicity group.

Table S5. CYP1A2 Nomenclature Translations and Diplotypes Identified Per Ethnicity Group

Diplotype	New Nomenclature	Ethnicity Group, *n* (%)					Total
		Asian	Black	White	Mixed or Multiple	Other	
*1/*1	*1/*1	2 (0.4%)	1 (0.2%)	2 (0.4%)	-	2 (0.4%)	7 (1.5%)
*1A/*1A	*1/*1	12 (2.6%)	3 (0.7%)	21 (4.6%)	3 (0.7%)	1 (0.2%)	40 (8.8%)
*1/*1F	*1/*30	1 (0.2%)	2 (0.4%)	7 (1.5%)	1 (0.2%)	-	11 (2.4%)
*1/*30	*1/*30	1 (0.2%)	2 (0.4%)	13 (2.9%)	2 (0.4%)	1 (0.2%)	19 (4.2%)
*1A/*1F	*1/*30	23 (5.1%)	16 (3.5%)	91 (20.1%)	3 (0.7%)	8 (1.8%)	141 (31.1%)
*1C/*1F	*1/*30	-	6 (1.3%)	2 (0.4%)	2 (0.2%)	2 (0.4%)	12 (2.6%)
*1F/*1F	*30/*30	19 (4.2%)	3 (0.7%)	138 (30.5%)	7 (1.5%)	2 (0.4%)	169 (37.3%)
*1K/*1F	*30/*30	2 (0.4%)	-	-	-	-	2 (0.4%)
*1L/*1F	*30/*30	4 (0.9%)	2 (0.4%)	5 (1.1%)	2 (0.4%)	2 (0.4%)	15 (3.3%)
*1L/*1L	*30/*30	1 (0.2%)	5 (1.1%)	1 (0.2%)	-	-	7 (1.5%)
*30/*30	*30/*30	2 (0.4%)	4 (0.9%)	22 (4.9%)	1 (0.2%)	1 (0.2%)	30 (6.6%)

Note: As of the change in CYP1A2 nomenclature (published 16-Dec-2024), *1A and *1C have been transitioned to the *1 core allele; *1F, *1K, and *1L have been transitioned under the new *30 core allele. For participants genotyped before this change, star alleles have been manually updated based on PharmVar and PharmGKB guidelines as of 22-Jan-2025.

Table S6. CYP3A4 Diplotypes Identified Per Ethnicity Group and Associated Metaboliser Status

Diplotype	Metaboliser Status	Ethnicity Group, *n* (%)					Total
		Asian	Black	White	Mixed or Multiple	Other	
*1/*1	Normal	67 (14.8%)	44 (9.7%)	265 (58.5%)	21 (4.6%)	17 (3.8%)	414 (91.4%)
*1/*22	Intermediate	-	-	36 (7.9%)	-	2 (0.4%)	38 (8.4%)
*22/*22	Poor	-	-	1 (0.2%)	-	-	1 (0.2%)

Table S7. CYP2D6 Diplotpes Identified Per Ethnicity Group and Associated Metaboliser Status

Diplotype	Metaboliser Status	Ethnicity Group, *n* (%)					Total
		Asian	Black	White	Mixed or Multiple	Other	
*1/*1	Normal	8 (1.8%)	5 (1.1%)	36 (7.9%)	-	1 (0.2%)	11.0%
*1/*10	Normal	6 (1.3%)	2 (0.4%)	4 (0.9%)	1 (0.2%)	1 (0.2%)	3.1%
*1/*13+*1	Normal	-	-	2 (0.4%)	-	-	0.2%
*1/*17	Normal	-	1 (0.2%)	-	3 (0.7%)	-	0.9%
*1/*1X2	Ultrarapid	-	-	2 (0.4%)	-	-	0.4%
*1/*2	Normal	10 (2.2%)	4 (0.9%)	34 (7.5%)	2 (0.4%)	6 (1.3%)	12.4%
*1/*29	Normal	-	1 (0.2%)	-	-	-	0.2%
*1/*2x2	Ultrarapid	2 (0.4%)	2 (0.4%)	2 (0.4%)	-	1 (0.2%)	1.5%
*1/*3	Intermediate	-	-	4 (0.9%)	-	-	0.9%
*1/*36+*10	Normal	3 (0.7%)	-	-	-	-	0.7%
*1/*4	Intermediate	4 (0.9%)	-	31 (6.8%)	-	-	7.7%
*1/*41	Normal	7 (1.5%)	1 (0.2%)	24 (5.3%)	-	3 (0.7%)	7.7%
*1/*41x2	Normal	1 (0.2%)	-	-	-	-	0.2%
*1/*4x2	Intermediate	-	1 (0.2%)	-	-	-	0.2%
*1/*5	Intermediate	1 (0.2%)	1 (0.2%)	6 (1.3%)	-	1 (0.2%)	2.0%
*1/*6	Intermediate	-	-	5 (1.1%)	-	-	1.1%
*1/*68+*4	Intermediate	1 (0.2%)	-	12 (2.6%)	1 (0.2%)	-	3.1%
*1/*7	Intermediate	1 (0.2%)	-	-	-	-	0.2%
*1/*9	Normal	-	1 (0.2%)	7 (1.5%)	-	-	1.8%
*10/*36+*10	Intermediate	1 (0.2%)	-	-	-	-	0.2%
*11/*5	Poor	-	-	1 (0.2%)	-	-	0.2%
*13+*1/*4	Intermediate	1 (0.2%)	-	-	-	-	0.2%
*14/*41	Intermediate	-	-	1 (0.2%)	-	-	0.2%
*15/*29	Intermediate	-	1 (0.2%)	-	-	-	0.2%
*17/*17	Intermediate	-	3 (0.7%)	-	1 (0.2%)	-	0.9%
*17/*29	Intermediate	-	1 (0.2%)	-	-	1 (0.2%)	0.4%
*17/*41	Intermediate	-	2 (0.4%)	-	-	-	0.4%
*17/*4N+*4	Intermediate	-	-	1 (0.2%)	-	-	0.2%
*17/*4x2	Intermediate	-	1 (0.2%)	-	-	-	0.2%
*1x2/*2	Ultrarapid	-	-	1 (0.2%)	-	-	0.2%
*1x2/*41	Normal	-	-	1 (0.2%)	-	-	0.2%
*2/*10	Normal	1 (0.2%)	-	1 (0.2%)	1 (0.2%)	-	0.7%
*2/*17	Normal	-	2 (0.4%)	-	-	1 (0.2%)	0.7%
*2/*2	Normal	5 (1.1%)	3 (0.7%)	9 (2.0%)	4 (0.9%)	-	4.6%
*2/*2x2	Ultrarapid	-	1 (0.2%)	-	-	-	0.2%
*2/*3	Intermediate	-	-	2 (0.4%)	-	-	0.4%
*2/*36+*10	Normal	2 (0.4%)	-	-	-	-	0.4%
*2/*4	Intermediate	1 (0.2%)	-	28 (6.2%)	-	1 (0.2%)	6.6%
*2/*41	Normal	6 (1.3%)	-	21 (4.6%)	-	1 (0.2%)	6.2%
*2/*4N+*4	Intermediate	-	-	1 (0.2%)	-	-	0.2%

Table S7. CYP2D6 Diplotpes Identified Per Ethnicity Group and Associated Metaboliser Status

Diplotype	Metaboliser Status	Ethnicity Group, *n* (%)					Total
		Asian	Black	White	Mixed or Multiple	Other	
*2/*4x2	Intermediate	-	1 (0.2%)	-	-	-	0.2%
*2/*5	Intermediate	1 (0.2%)	1 (0.2%)	3 (0.7%)	1 (0.2%)	-	1.3%
*2/*68+*4	Intermediate	-	-	5 (1.1%)	2 (0.4%)	2 (0.4%)	2.0%
*2/*9	Normal	-	-	2 (0.4%)	1 (0.2%)	-	0.7%
*29/*41	Intermediate	-	1 (0.2%)	-	-	-	0.2%
*2x2/*17	Ultrarapid	-	2 (0.4%)	-	-	-	0.4%
*2x2/*4	Normal	-	-	1 (0.2%)	-	-	0.2%
*2x2/*41	Normal	-	-	1 (0.2%)	-	-	0.2%
*2x2/*4N+*4	Normal	-	-	1 (0.2%)	-	-	0.2%
*3/*13	Poor	-	-	1 (0.2%)	-	-	0.2%
*3/*3	Poor	-	-	2 (0.4%)	-	-	0.4%
*3/*4	Poor	-	-	2 (0.4%)	-	-	0.4%
*3/*41	Intermediate	-	-	1 (0.2%)	-	-	0.2%
*3/*68+*4	Poor	-	-	1 (0.2%)	-	-	0.2%
*4/*10	Intermediate	1 (0.2%)	-	2 (0.4%)	1 (0.2%)	-	0.9%
*4/*17	Intermediate	-	1 (0.2%)	-	-	-	0.2%
*4/*29	Intermediate	-	1 (0.2%)	-	-	-	0.2%
*4/*4	Poor	-	-	5 (1.1%)	-	-	1.1%
*4/*41	Intermediate	-	-	12 (2.6%)	3 (0.7%)	-	3.3%
*4/*6	Poor	-	-	1 (0.2%)	-	-	0.2%
*4/*68+*4	Poor	1 (0.2%)	1 (0.2%)	6 (1.3%)	-	-	1.8%
*4/*9	Intermediate	-	-	3 (0.7%)	-	-	0.7%
*41/*41	Intermediate	3 (0.7%)	-	3 (0.7%)	-	-	1.3%
*41/*68+*4	Intermediate	-	-	1 (0.2%)	-	-	0.2%
*4N+*4/*5	Poor	-	-	1 (0.2%)	-	-	0.2%
*4x2/*29	Intermediate	-	1 (0.2%)	-	-	-	0.2%
*5/*17	Intermediate	-	1 (0.2%)	-	-	-	0.2%
*5/*29	Intermediate	-	1 (0.2%)	-	-	-	0.2%
*5/*41	Intermediate	-	-	4 (0.9%)	-	-	0.9%
*5/*68+*4	Poor	-	-	2 (0.4%)	-	-	0.4%
*6/*41	Intermediate	-	-	1 (0.2%)	-	-	0.2%
*9/*41	Intermediate	-	-	1 (0.2%)	-	-	0.2%
*9/*9	Intermediate	-	-	1 (0.2%)	-	-	0.2%

Table S8. Prevalence of Each PharmGKB Level 1A Non-Normal Function Variant

Gene	Allele	Action	Number of Carriers (%)
CYP2D6	*1xN	Increased function	4 (0.9%)
	*3	No function	13 (2.9%)
	*4	No function	109 (24.1%)
	*5	No function	25 (5.5%)
	*6	No function	7 (1.5%)
	*10	Decreased function	22 (4.9%)
	*14	Decreased function	1 (0.2%)
	*17	Decreased function	21 (4.6%)
	*41	Decreased function	98 (21.6%)
CYP3A4	*20	No function	*Not on panel – unable to detect*
	*22	Decreased function	39 (8.6%)

Alleles taken from the PharmGKB (CYP2D6, CYP3A4) (correct as of December 2024)
Note: *1xN refers to a duplication of *1 of any number (e.g. *1x2, *1x3, etc.)
Abbreviations: PharmGKB = Pharmacogenomics Knowledgebase

Table S9. Effect of Pharmacogenetic Variation in *CYP1A2*, *CYP2D6*, and *CYP3A4* on Self-Reported Adverse Drug Reactions to Antipsychotic Medications

**Variable**		**Mean Difference**	**95% CI**	***p***
CYP2D6 [ref = NM]	Poor Metaboliser	5.124	-1.973, 12.221	0.158
	Intermediate Metaboliser	1.173	-1.977, 4.323	0.466
	Ultrarapid Metaboliser	-11.069	-18.886, -3.252	0.00575
CYP1A2 [ref = *1/*1]	*1/*30	0.401	-5.202, 6.004	0.888
	*30/*30	2.762	-2.823, 8.347	0.333
CYP3A4 [ref = NM]	Reduced Metaboliser	3.696	-2.494, 9.887	0.243
Age [continuous]		-0.015	-0.015, 0.088	0.776
Sex [ref = Female]	Male	-3.698	-6.950, -0.445	0.0264
Ethnicity [ref = White]	Asian or Asian British	1.142	-3.101, 5.385	0.598
	Black, Black British, Caribbean or African	5.981	0.596, 11.366	0.0300
	Mixed or multiple ethnic groups	-4.335	-9.851, 1.181	0.124
	Other ethnic group	4.346	-3.719, 12.410	0.291
Diagnosis [ref = schizophrenia]	Bipolar Disorder	-0.801	-5.693, 4.091	0.748
	Other psychotic disorder	0.103	-4.015, -4.015	0.961
	Other psychiatric disorder	-0.488	-6.585, 5.609	0.875
Antipsychotic medication [ref = aripiprazole]	Clozapine	-6.032	-11.938, -0.127	0.0459
	Olanzapine	-3.550	-8.704, 1.603	0.178
	Quetiapine	1.888	-3.491, 7.266	0.492
	Other antipsychotic (monotherapy)	-0.331	-5.378, 4.716	0.898
	Multiple antipsychotics	1.114	-3.814, 6.042	0.658
CYP3A4 inducer [ref = no]	Yes	-4.605	-17.999, 8.789	0.501
CYP3A4 inhibitor [ref = no]	Yes	-1.663	-9.057, 5.731	0.660
CYP1A2 inducer [ref = no]	Yes	3.501	-0.277, 7.279	0.0700
Antipsychotic dose [continuous]		-0.532	-5.961, 4.896	0.848
Antidepressant dose [continuous]		3.863	0.090, 7.636	0.0454
Mood stabiliser dose [continuous]		2.489	-2.726, 7.703	0.350

Abbreviations: CI = confidence interval; NM = normal metaboliser; ref = reference category

Table S10. Effect of Pharmacogenetic Variation in *CYP1A2*, *CYP2D6*, and *CYP3A4* on Self-Reported Extra Pyramidal Adverse Reactions to Antipsychotic Medications (LUNSERS Extrapyramidal Domain)

**Variable**		**Mean Difference**	**95% CI**	***p***
CYP2D6 [ref = NM]	Poor Metaboliser	6.754	-0.947, 14.455	0.0864
	Intermediate Metaboliser	0.193	-3.422, 3.807	0.917
	Ultrarapid Metaboliser	-12.943	-21.899, -3.987	0.00484
CYP1A2 [ref = *1/*1]	*1/*30	2.180	-4.379, 8.739	0.515
	*30/*30	4.771	-1.874, 11.416	0.160
CYP3A4 [ref = NM]	Reduced Metaboliser	4.825	-2.033, 11.683	0.169
Age [continuous]		0.0974	-0.0229, 0.218	0.113
Sex [ref = Female]	Male	-2.942	-6.763, 0.879	0.132
Ethnicity [ref = White]	Asian or Asian British	5.190	-0.417, 10.796	0.0703
	Black, Black British, Caribbean or African	6.515	0.560, 12.469	0.0326
	Mixed or multiple ethnic groups	-6.790	-13.032, -0.547	0.0336
	Other ethnic group	7.833	-2.367, 18.032	0.133
Diagnosis [ref = schizophrenia]	Bipolar Disorder	-3.240	-9.182, 2.701	0.286
	Other psychotic disorder	-0.347	-5.087, 4.392	0.886
	Other psychiatric disorder	-4.113	-10.795, 2.570	0.228
Antipsychotic medication [ref = aripiprazole]	Clozapine	-5.629	-12.531, 1.273	0.111
	Olanzapine	-3.142	-9.067, 2.782	0.299
	Quetiapine	-2.099	-8.345, 4.146	0.510
	Other antipsychotic (monotherapy)	-1.545	-7.637, 4.548	0.619
	Multiple antipsychotics	0.754	-5.358, 6.867	0.809
CYP3A4 inducer [ref = no]	Yes	-4.043	-16.245, 8.159	0.516
CYP3A4 inhibitor [ref = no]	Yes	-1.598	-9.506, 6.310	0.692
CYP1A2 inducer [ref = no]	Yes	2.768	-1.590, 7.126	0.214
Antipsychotic dose [continuous]		-1.883	-8.196, 4.429	0.559
Antidepressant dose [continuous]		1.723	-2.521, 5.967	0.427
Mood stabiliser dose [continuous]		2.527	-3.091, 8.145	0.378

Abbreviations: CI = confidence interval; NM = normal metaboliser; ref = reference category

Table S11. Effect of Pharmacogenetic Variation in *CYP1A2*, *CYP2D6*, and *CYP3A4* on Self-Reported Anticholinergic Adverse Reactions to Antipsychotic Medications (LUNSERS Anticholinergic Domain)

**Variable**		**Mean Difference**	**95% CI**	***p***
CYP2D6 [ref = NM]	Poor Metaboliser	6.708	-0.803, 14.219	0.0808
	Intermediate Metaboliser	1.373	-2.521, 5.267	0.490
	Ultrarapid Metaboliser	-3.617	-13.910, 6.675	0.491
CYP1A2 [ref = *1/*1]	*1/*30	2.106	-4.207, 8.420	0.514
	*30/*30	5.041	-1.224, 11.306	0.116
CYP3A4 [ref = NM]	Reduced Metaboliser	4.096	-2.837, 11.028	0.248
Age [continuous]		0.157	0.0356, 0.279	0.0116
Sex [ref = Female]	Male	-5.353	-9.333, -1.374	0.00867
Ethnicity [ref = White]	Asian or Asian British	1.405	-3.769, 6.578	0.595
	Black, Black British, Caribbean or African	2.698	-3.503, 8.898	0.394
	Mixed or multiple ethnic groups	0.855	-7.557, 9.266	0.842
	Other ethnic group	3.576	-6.033, 13.185	0.466
Diagnosis [ref = schizophrenia]	Bipolar Disorder	-0.309	-6.385, 5.768	0.921
	Other psychotic disorder	-0.142	-5.161, 4.877	0.956
	Other psychiatric disorder	-2.994	-10.465, 4.476	0.432
Antipsychotic medication [ref = aripiprazole]	Clozapine	-1.833	-8.825, 5.159	0.608
	Olanzapine	-2.483	-8.500, 3.535	0.419
	Quetiapine	6.645	0.347, 12.943	0.0392
	Other antipsychotic (monotherapy)	2.544	-3.659, 8.746	0.422
	Multiple antipsychotics	4.382	-1.899, 10.663	0.172
CYP3A4 inducer [ref = no]	Yes	-2.253	-16.822, 12.316	0.762
CYP3A4 inhibitor [ref = no]	Yes	-1.795	-10.142, 6.551	0.674
CYP1A2 inducer [ref = no]	Yes	5.641	0.952, 10.330	0.0188
Antipsychotic dose [continuous]		4.749	-1.907, 11.405	0.163
Antidepressant dose [continuous]		2.467	-2.011, 6.944	0.281
Mood stabiliser dose [continuous]		2.213	-4.060, 8.486	0.490

Abbreviations: CI = confidence interval; NM = normal metaboliser; ref = reference category

Table S12. Effect of Pharmacogenetic Variation in *CYP1A2*, *CYP2D6*, and *CYP3A4* on Self-Reported Other Autonomic Adverse Reactions to Antipsychotic Medications (LUNSERS Other Autonomic Domain)

**Variable**		**Mean Difference**	**95% CI**	***p***
CYP2D6 [ref = NM]	Poor Metaboliser	3.716	-5.570, 13.003	0.433
	Intermediate Metaboliser	-0.0152	-4.076, 4.045	0.994
	Ultrarapid Metaboliser	-15.470	-22.541, -8.398	0.0000223
CYP1A2 [ref = *1/*1]	*1/*30	-2.223	-9.324, 4.878	0.540
	*30/*30	-0.357	-7.270, 6.557	0.919
CYP3A4 [ref = NM]	Reduced Metaboliser	-2.889	-10.020, 4.242	0.428
Age [continuous]		-0.112	-0.241, 0.0176	0.0912
Sex [ref = Female]	Male	-6.101	-10.394, -1.808	0.00558
Ethnicity [ref = White]	Asian or Asian British	-2.711	-8.377, 2.955	0.349
	Black, Black British, Caribbean or African	4.470	-2.351, 11.292	0.200
	Mixed or multiple ethnic groups	-8.652	-15.443, -1.860	0.0129
	Other ethnic group	6.232	-5.554, 18.018	0.301
Diagnosis [ref = schizophrenia]	Bipolar Disorder	-3.658	-9.913, 2.596	0.252
	Other psychotic disorder	1.534	-6.385, 9.454	0.704
	Other psychiatric disorder	-3.072	-8.041, 1.897	0.226
Antipsychotic medication [ref = aripiprazole]	Clozapine	-6.701	-13.806, 0.405	0.0653
	Olanzapine	-4.749	-11.239, 1.741	0.152
	Quetiapine	2.732	-4.366, 9.830	0.451
	Other antipsychotic (monotherapy)	-2.248	-8.533, 4.037	0.484
	Multiple antipsychotics	3.097	-3.437, 9.631	0.353
CYP3A4 inducer [ref = no]	Yes	-2.100	-18.364, 14.163	0.800
CYP3A4 inhibitor [ref = no]	Yes	-2.619	-10.940, 5.702	0.538
CYP1A2 inducer [ref = no]	Yes	2.889	-2.019, 7.797	0.249
Antipsychotic dose [continuous]		2.210	-4.472, 8.893	0.517
Antidepressant dose [continuous]		4.427	-0.368, 9.221	0.0710
Mood stabiliser dose [continuous]		0.897	-4.805, 6.599	0.758

Abbreviations: CI = confidence interval; NM = normal metaboliser; ref = reference category

Table S13. Effect of Pharmacogenetic Variation in *CYP1A2*, *CYP2D6*, and *CYP3A4* on Self-Reported Allergic Adverse Reactions to Antipsychotic Medications (LUNSERS Allergic Reactions Domain)

**Variable**		**Mean Difference**	**95% CI**	***p***
CYP2D6 [ref = NM]	Poor Metaboliser	2.520	-5.024, 10.064	0.513
	Intermediate Metaboliser	1.503	-2.296, 5.302	0.439
	Ultrarapid Metaboliser	-3.161	-14.834, 8.513	0.596
CYP1A2 [ref = *1/*1]	*1/*30	-1.182	-8.349, 5.986	0.747
	*30/*30	-0.258	-7.343, 6.828	0.943
CYP3A4 [ref = NM]	Reduced Metaboliser	4.388	-3.239, 12.015	0.260
Age [continuous]		-0.0572	-0.180, 0.0657	0.362
Sex [ref = Female]	Male	-4.544	-8.549, -0.538	0.0267
Ethnicity [ref = White]	Asian or Asian British	0.101	-4.997, 5.200	0.969
	Black, Black British, Caribbean or African	3.600	-2.765, 9.964	0.268
	Mixed or multiple ethnic groups	-3.200	-11.664, 5.264	0.459
	Other ethnic group	-0.259	-9.975, 9.456	0.958
Diagnosis [ref = schizophrenia]	Bipolar Disorder	0.687	-5.147, 6.521	0.818
	Other psychotic disorder	-0.878	-5.771, 4.015	0.725
	Other psychiatric disorder	0.123	-7.729, 7.974	0.976
Antipsychotic medication [ref = aripiprazole]	Clozapine	-1.164	-7.851, 5.523	0.733
	Olanzapine	-0.723	-6.186, 4.740	0.795
	Quetiapine	3.219	-3.277, 9.715	0.332
	Other antipsychotic (monotherapy)	4.909	-1.508, 11.326	0.135
	Multiple antipsychotics	4.484	-1.866, 10.833	0.167
CYP3A4 inducer [ref = no]	Yes	-0.075	-17.475, 17.326	0.993
CYP3A4 inhibitor [ref = no]	Yes	-0.442	-8.215, 7.330	0.911
CYP1A2 inducer [ref = no]	Yes	2.984	-1.950, 7.917	0.237
Antipsychotic dose [continuous]		0.674	-5.584, 6.932	0.833
Antidepressant dose [continuous]		-1.718	-5.999, 2.562	0.432
Mood stabiliser dose [continuous]		0.558	-5.922, 7.038	0.866

Abbreviations: CI = confidence interval; NM = normal metaboliser; ref = reference category

Table S14. Effect of Pharmacogenetic Variation in *CYP1A2*, *CYP2D6*, and *CYP3A4* on Self-Reported Psychic Adverse Reactions to Antipsychotic Medications (LUNSERS Psychic Domain)

Variable		Mean Difference	95% CI	*p*
CYP2D6 [ref = NM]	Poor Metaboliser	5.342	-4.074, 14.758	0.267
	Intermediate Metaboliser	1.271	-2.954, 5.496	0.556
	Ultrarapid Metaboliser	-15.559	-28.767, -2.351	0.0214
CYP1A2 [ref = *1/*1]	*1/*30	1.431	-6.006, 8.867	0.706
	*30/*30	4.258	-3.224, 11.740	0.265
CYP3A4 [ref = NM]	Reduced Metaboliser	4.261	-3.707, 12.229	0.295
Age [continuous]		-0.026	-0.171, 0.119	0.726
Sex [ref = Female]	Male	-3.571	-7.797, 0.655	0.0985
Ethnicity [ref = White]	Asian or Asian British	1.653	-4.190, 7.496	0.580
	Black, Black British, Caribbean or African	4.910	-2.634, 12.453	0.203
	Mixed or multiple ethnic groups	-1.758	-9.732, 6.217	0.666
	Other ethnic group	3.743	-6.752, 14.238	0.485
Diagnosis [ref = schizophrenia]	Bipolar Disorder	1.563	-5.024, 8.151	0.642
	Other psychotic disorder	0.424	-5.034, 5.882	0.879
	Other psychiatric disorder	-0.616	-8.429, 7.198	0.877
Antipsychotic medication [ref = aripiprazole]	Clozapine	-11.759	-19.976, -3.542	0.00527
	Olanzapine	-6.493	-13.789, 0.803	0.0818
	Quetiapine	-0.273	-7.298, 6.752	0.939
	Other antipsychotic (monotherapy)	-3.919	-10.426, 2.588	0.238
	Multiple antipsychotics	-4.640	-11.193, 1.914	0.166
CYP3A4 inducer [ref = no]	Yes	-6.226	-21.480, 9.027	0.424
CYP3A4 inhibitor [ref = no]	Yes	-5.067	-14.613, 4.478	0.299
CYP1A2 inducer [ref = no]	Yes	3.189	-1.546, 7.925	0.188
Antipsychotic dose [continuous]		-4.405	-11.476, 2.667	0.223
Antidepressant dose [continuous]		10.483	5.587, 15.379	0.0000330
Mood stabiliser dose [continuous]		4.660	-3.132, 12.453	0.242

Abbreviations: CI = confidence interval; NM = normal metaboliser; ref = reference category

Table S15. Effect of Pharmacogenetic Variation in *CYP1A2*, *CYP2D6*, and *CYP3A4* on Self-Reported Hormonal Adverse Reactions to Antipsychotic Medications (LUNSERS Hormonal Domain)

Variable		Mean Difference	95% CI	*p*
CYP2D6 [ref = NM]	Poor Metaboliser	5.538	-1.177, 12.254	0.107
	Intermediate Metaboliser	2.590	-0.865, 6.046	0.143
	Ultrarapid Metaboliser	-10.110	-18.111, -2.109	0.0136
CYP1A2 [ref = *1/*1]	*1/*30	-2.336	-8.268, 3.595	0.441
	*30/*30	-0.331	-6.094, 5.431	0.910
CYP3A4 [ref = NM]	Reduced Metaboliser	4.311	-2.510, 11.131	0.216
Age [continuous]		-0.168	-0.293, -0.0429	0.00877
Sex [ref = Female]	Male	-1.271	-4.954, 2.412	0.499
Ethnicity [ref = White]	Asian or Asian British	0.990	-3.920, 5.900	0.693
	Black, Black British, Caribbean or African	8.789	1.867, 15.710	0.0132
	Mixed or multiple ethnic groups	-3.138	-10.457, 4.181	0.401
	Other ethnic group	2.482	-5.931, 10.896	0.563
Diagnosis [ref = schizophrenia]	Bipolar Disorder	0.492	-5.172, 6.155	0.865
	Other psychotic disorder	3.041	-1.644, 7.727	0.204
	Other psychiatric disorder	3.279	-3.907, 10.466	0.372
Antipsychotic medication [ref = aripiprazole]	Clozapine	-3.621	-9.829, 2.586	0.254
	Olanzapine	-2.842	-8.010, 2.326	0.282
	Quetiapine	3.658	-1.975, 9.291	0.204
	Other antipsychotic (monotherapy)	4.273	-1.298, 9.844	0.133
	Multiple antipsychotics	4.935	-0.810, 10.680	0.0930
CYP3A4 inducer [ref = no]	Yes	-7.107	-22.552, 8.338	0.368
CYP3A4 inhibitor [ref = no]	Yes	4.336	-5.031, 13.703	0.365
CYP1A2 inducer [ref = no]	Yes	4.160	-0.122, 8.443	0.0576
Antipsychotic dose [continuous]		-1.189	-7.307, 4.928	0.703
Antidepressant dose [continuous]		0.715	-3.635, 5.066	0.747
Mood stabiliser dose [continuous]		3.042	-3.532, 9.615	0.365

Abbreviations: CI = confidence interval; NM = normal metaboliser; ref = reference category

Table S16. Effect of Pharmacogenetic Variation in *CYP1A2*, *CYP2D6*, and *CYP3A4* on Self-Reported Miscellaneous Adverse Reactions to Antipsychotic Medications (LUNSERS Miscellaneous Domain)

**Variable**		**Mean Difference**	**95% CI**	***p***
CYP2D6 [ref = NM]	Poor Metaboliser	3.915	-4.229, 12.059	0.347
	Intermediate Metaboliser	1.718	-1.697, 5.134	0.325
	Ultrarapid Metaboliser	-7.570	-17.445, 2.305	0.134
CYP1A2 [ref = *1/*1]	*1/*30	1.159	-4.483, 6.801	0.687
	*30/*30	2.131	-3.406, 7.668	0.451
CYP3A4 [ref = NM]	Reduced Metaboliser	1.057	-5.093, 7.207	0.736
Age [continuous]		-0.088	-0.204, 0.0280	0.138
Sex [ref = Female]	Male	-5.815	-9.248, -2.381	0.000979
Ethnicity [ref = White]	Asian or Asian British	-0.0869	-4.734, 4.560	0.971
	Black, Black British, Caribbean or African	9.831	4.068, 15.595	0.000900
	Mixed or multiple ethnic groups	-7.987	-14.941, -1.033	0.0249
	Other ethnic group	1.884	-6.379, 10.147	0.655
Diagnosis [ref = schizophrenia]	Bipolar Disorder	-1.694	-6.667, 3.279	0.505
	Other psychotic disorder	0.816	-3.446, 5.077	0.708
	Other psychiatric disorder	3.107	-3.837, 10.051	0.381
Antipsychotic medication [ref = aripiprazole]	Clozapine	-2.215	-8.732, 4.303	0.506
	Olanzapine	1.512	-4.268, 7.292	0.608
	Quetiapine	4.533	-1.336, 10.402	0.131
	Other antipsychotic (monotherapy)	0.0253	-4.994, 5.045	0.992
	Multiple antipsychotics	3.731	-1.815, 9.276	0.188
CYP3A4 inducer [ref = no]	Yes	2.160	-15.607, 19.926	0.812
CYP3A4 inhibitor [ref = no]	Yes	-0.583	-7.257, 6.090	0.864
CYP1A2 inducer [ref = no]	Yes	1.588	-2.188, 5.363	0.410
Antipsychotic dose [continuous]		-0.584	-6.267, 5.099	0.840
Antidepressant dose [continuous]		1.605	-2.058, 5.269	0.391
Mood stabiliser dose [continuous]		-0.187	-5.476, 5.102	0.945

Abbreviations: CI = confidence interval; NM = normal metaboliser; ref = reference category

Table S17. Effect of Pharmacogenetic Variation in *CYP1A2*, *CYP2D6*, and *CYP3A4* on Self-Reported Health-Related Quality of Life

Variable		Mean Difference	95% CI	*p*
CYP2D6 [ref = NM]	Poor Metaboliser	0.0235	-0.0971, 0.1441	0.703
	Intermediate Metaboliser	-0.0008	-0.0571, 0.0555	0.978
	Ultrarapid Metaboliser	0.0611	-0.1179, 0.2401	0.503
CYP1A2 [ref = *1/*1]	*1/*30	-0.0863	-0.1806, 0.0081	0.0731
	*30/*30	-0.0803	-0.1734, 0.0129	0.0914
CYP3A4 [ref = NM]	Reduced Metaboliser	-0.0779	-0.1759, 0.0201	0.119
Age [continuous]		-0.0013	-0.0033, 0.0006	0.188
Sex [ref = Female]	Male	0.0806	0.0227, 0.1384	0.00633
Ethnicity [ref = White]	Asian or Asian British	-0.0531	-0.1339, 0.0277	0.198
	Black, Black British, Caribbean or African	-0.0569	-0.1541, 0.0403	0.251
	Mixed or multiple ethnic groups	0.0023	-0.1278, 0.1324	0.973
	Other ethnic group	-0.0688	-0.2050, 0.0674	0.322
Diagnosis [ref = schizophrenia]	Bipolar Disorder	0.0107	-0.0796, 0.1011	0.816
	Other psychotic disorder	-0.0338	-0.1060, 0.0384	0.359
	Other psychiatric disorder	-0.0418	-0.1511, 0.0675	0.453
Antipsychotic medication [ref = aripiprazole]	Clozapine	0.0695	-0.0480, 0.1870	0.247
	Olanzapine	0.0876	-0.0072, 0.1823	0.0700
	Quetiapine	-0.0458	-0.1393, 0.0477	0.337
	Other antipsychotic (monotherapy)	-0.0058	-0.0941, 0.0825	0.897
	Multiple antipsychotics	-0.0377	-0.1311, 0.0557	0.429
CYP3A4 inducer [ref = no]	Yes	0.0941	-0.1111, 0.2993	0.369
CYP3A4 inhibitor [ref = no]	Yes	0.1431	0.0299, 0.2562	0.0132
CYP1A2 inducer [ref = no]	Yes	-0.0174	-0.0821, 0.0473	0.598
Antipsychotic dose [continuous]		-0.0274	-0.1201, 0.0654	0.563
Antidepressant dose [continuous]		-0.1567	-0.2199, -0.0935	0.00000118
Mood stabiliser dose [continuous]		-0.0158	-0.1089, 0.0773	0.740

Abbreviations: CI = confidence interval; NM = normal metaboliser; ref = reference category

Table S18. Effect of Pharmacogenetic Variation in *CYP1A2*, *CYP2D6*, and *CYP3A4* on Prescribed Antipsychotic Medication Dose

Variable		Mean Difference	95% CI	*p*
CYP2D6 [ref = NM]	Poor Metaboliser	-9.241	-20.724, 2.241	0.115
	Intermediate Metaboliser	-1.065	-6.541, 4.410	0.703
	Ultrarapid Metaboliser	1.073	-17.828, 19.974	0.911
CYP1A2 [ref = *1/*1]	*1/*30	2.291	-7.750, 12.332	0.655
	*30/*30	-4.664	-14.557, 5.229	0.356
CYP3A4 [ref = NM]	Reduced Metaboliser	-7.870	-18.356, 2.616	0.142
Age [continuous]		-0.212	-0.400, -0.024	0.0279
Sex [ref = Female]	Male	6.510	1.029, 11.990	0.0204
Ethnicity [ref = White]	Asian or Asian British	-6.943	-14.579, 0.693	0.0755
	Black, Black British, Caribbean or African	-1.266	-11.452, 8.919	0.808
	Mixed or multiple ethnic groups	-9.535	-22.570, 3.499	0.152
	Other ethnic group	-7.859	-21.892, 6.173	0.273
Diagnosis [ref = schizophrenia]	Bipolar Disorder	-5.195	-14.874, 4.485	0.293
	Other psychotic disorder	-3.330	-10.962, 4.301	0.393
	Other psychiatric disorder	-17.060	-27.321, -6.800	0.00121
Antipsychotic medication [ref = aripiprazole]	Clozapine	-22.869	-33.120, -12.618	0.0000155
	Olanzapine	4.154	-5.223, 13.531	0.386
	Quetiapine	-14.400	-22.802, -5.997	0.000853
	Other antipsychotic (monotherapy)	-23.615	-32.207, -15.024	0.000000118
	Multiple antipsychotics	13.477	2.774, 24.181	0.0140
CYP3A4 inducer [ref = no]	Yes	10.559	-11.341, 32.460	0.345
CYP3A4 inhibitor [ref = no]	Yes	2.414	-9.046, 13.874	0.680
CYP1A2 inducer [ref = no]	Yes	7.111	0.507, 13.714	0.0354
Antidepressant dose [continuous]		1.579	-4.927, 8.086	0.635
Mood stabiliser dose [continuous]		7.968	-3.158, 19.094	0.161

Abbreviations: CI = confidence interval; NM = normal metaboliser; ref = reference category

Table S19. Demographic Characteristics of the Psychosis Diagnosis-Only Subgroup of the Pharmacogenetics in Mental Health Study

**Sample size,** *N*	400
**Age**, mean years (SD)	43.5 (14.4)
Age range, years	18 – 82
**Sex**, *n* (%)	
Male	226 (56.5%)
Female	174 (43.5%)
**Ethnicity**, *n* (%)	
Asian or Asian British	59 (14.8)
Black, Black British, Caribbean or African	44 (11.0%)
White	259 (64.8%)
Mixed or multiple ethnic groups	20 (5.0%)
Other ethnic group	18 (4.5%)
**Duration of illness**[1], mean years (SD)	11.6 (11.2)
Duration of illness range, years	0 – 50
**Location recruited**, *n* (%)	
Community	242 (60.5%)
Inpatient	129 (32.3%)
Missing	*29 (7.3%)*
**Antipsychotic(s) taken**[2], *n* (%)	
Aripiprazole	114 (28.5%)
Olanzapine	84 (21.0%)
Quetiapine	52 (13.0%)
Clozapine	73 (18.2%)
Risperidone	35 (8.8%)
Zuclopenthixol	30 (7.5%)
Amisulpride	23 (5.8%)
Paliperidone	20 (5.0%)
Lurasidone	18 (4.5%)
Flupentixol	16 (4.0%)
Haloperidol	10 (2.5%)
Cariprazine	2 (0.5%)
Promazine	1 (0.2%)
**Taking antidepressant(s)**, *n* (%)	147 (36.8%)
**Taking mood stabiliser(s)**, *n* (%)	125 (31.3%)

[1] Duration of illness calculated as the difference between the participants age at the time of the baseline assessment and the age they were diagnosed with their classified primary diagnosis. Data missing for 68 (17%) participants.
[2] In the case that an individual is taking multiple antipsychotics, all have been counted here
Abbreviations: SD = standard deviation; n = number of participants

Table S20. Cytochrome P450 (CYP) Enzyme Metaboliser Status Frequency in the Psychosis Diagnosis-Only Subgroup

Enzyme	Metaboliser Status/Diplotype Group	*n* (%)
**CYP2D6**	Poor Metaboliser	24 (6.0%)
	Intermediate Metaboliser	170 (42.5%)
	Normal Metaboliser	196 (49.0%)
	Ultrarapid Metaboliser	10 (2.5%)
**CYP1A2**	*1/*1	44 (11.0%)
	*1/*30	162 (40.5%)
	*30/*30	194 (48.5%)
**CYP3A4**	Intermediate Metaboliser	30 (7.5%)
	Normal Metaboliser	370 (92.5%)

Note: For CYP2D6, metaboliser statuses are classified after phenoconversion, where this is possible to account for.
Abbreviations: SD = standard deviation; n = number of participants

Table S21. Effect of Pharmacogenetic Variation in *CYP1A2*, *CYP2D6*, and *CYP3A4* on Self-Reported Adverse Drug Reactions to Antipsychotic Medications in Participants with a Diagnosis of Psychosis Only

Variable		Mean Difference	95% CI	*p*
CYP2D6 [ref = NM]	Poor Metaboliser	6.188	-1.174, 13.549	0.100
	Intermediate Metaboliser	1.179	-2.180, 4.538	0.492
	Ultrarapid Metaboliser	-11.319	-19.662, -2.977	0.00816
CYP1A2 [ref = *1/*1]	*1/*30	0.374	-5.526, 6.274	0.901
	*30/*30	3.280	-2.645, 9.205	0.279
CYP3A4 [ref = NM]	Reduced Metaboliser	6.242	-0.612, 13.095	0.0751
Age [continuous]		-0.026	-0.136, 0.083	0.636
Sex [ref = Female]	Male	-3.870	-7.416, -0.325	0.0330
Ethnicity [ref = White]	Asian or Asian British	2.332	-2.107, 6.771	0.304
	Black, Black British, Caribbean or African	6.569	1.147, 11.990	0.0181
	Mixed or multiple ethnic groups	-3.872	-9.651, 1.907	0.190
	Other ethnic group	3.649	-4.620, 11.917	0.388
Diagnosis [ref = schizophrenia]	Bipolar Disorder	-1.496	-6.428, 3.436	0.552
	Other psychotic disorder	-0.261	-4.434, 3.911	0.902
Antipsychotic medication [ref = aripiprazole]	Clozapine	-6.500	-12.539, -0.461	0.0356
	Olanzapine	-3.554	-8.981, 1.872	0.200
	Quetiapine	2.565	-3.380, 8.511	0.398
	Other antipsychotic (monotherapy)	-1.061	-6.340, 4.217	0.694
	Multiple antipsychotics	0.018	-5.228, 5.264	0.995
CYP3A4 inducer [ref = no]	Yes	-3.256	-17.751, 11.240	0.660
CYP3A4 inhibitor [ref = no]	Yes	-1.904	-9.196, 5.389	0.609
CYP1A2 inducer [ref = no]	Yes	3.099	-0.917, 7.116	0.131
Antipsychotic dose [continuous]		-0.455	-6.029, 5.120	0.873
Antidepressant dose [continuous]		4.488	0.699, 8.277	0.0208
Mood stabiliser dose [continuous]		2.657	-2.656, 7.971	0.328

Abbreviations: CI = confidence interval; NM = normal metaboliser; ref = reference category

Table S22. Effect of Pharmacogenetic Variation in *CYP1A2*, *CYP2D6*, and *CYP3A4* on Self-Reported Health-Related Quality of Life in Participants with a Diagnosis of Psychosis Only

Variable		Mean Difference	95% CI	*p*
CYP2D6 [ref = NM]	Poor Metaboliser	0.0124	-0.1113, 0.1361	0.844
	Intermediate Metaboliser	-0.0038	-0.0629, 0.0552	0.899
	Ultrarapid Metaboliser	0.0590	-0.1262, 0.2442	0.532
CYP1A2 [ref = *1/*1]	*1/*30	-0.0855	-0.1813, 0.0104	0.0807
	*30/*30	-0.0974	-0.1925, -0.0023	0.0447
CYP3A4 [ref = NM]	Reduced Metaboliser	-0.1359	-0.2446, -0.0272	0.0142
Age [continuous]		-0.0009	-0.0030, 0.0012	0.406
Sex [ref = Female]	Male	0.0907	0.0290, 0.1524	0.00395
Ethnicity [ref = White]	Asian or Asian British	-0.0762	-0.1611, 0.0087	0.0786
	Black, Black British, Caribbean or African	-0.0750	-0.1716, 0.0215	0.128
	Mixed or multiple ethnic groups	-0.0104	-0.1422, 0.1213	0.877
	Other ethnic group	-0.0271	-0.1665, 0.1123	0.703
Diagnosis [ref = schizophrenia]	Bipolar Disorder	0.0243	-0.0664, 0.1150	0.600
	Other psychotic disorder	-0.0301	-0.1015, 0.0412	0.408
Antipsychotic medication [ref = aripiprazole]	Clozapine	0.0631	-0.0545, 0.1808	0.293
	Olanzapine	0.0642	-0.0341, 0.1624	0.201
	Quetiapine	-0.0876	-0.1929, 0.0178	0.103
	Other antipsychotic (monotherapy)	-0.0228	-0.1143, 0.0687	0.625
	Multiple antipsychotics	-0.0276	-0.1236, 0.0684	0.573
CYP3A4 inducer [ref = no]	Yes	0.1321	-0.0854, 0.3496	0.234
CYP3A4 inhibitor [ref = no]	Yes	0.1769	0.0560, 0.2978	0.00413
CYP1A2 inducer [ref = no]	Yes	-0.0065	-0.0738, 0.0608	0.849
Antipsychotic dose [continuous]		-0.0249	-0.1200, 0.0701	0.607
Antidepressant dose [continuous]		-0.1510	-0.2177, -0.0842	0.00000933
Mood stabiliser dose [continuous]		-0.0247	-0.1192, 0.0699	0.609

Abbreviations: CI = confidence interval; NM = normal metaboliser; ref = reference category

Table S23. Effect of Pharmacogenetic Variation in *CYP1A2*, *CYP2D6*, and *CYP3A4* on Prescribed Antipsychotic Medication Dose in Participants with a Diagnosis of Psychosis Only

**Variable**		**Mean Difference**	**95% CI**	***p***
CYP2D6 [ref = NM]	Poor Metaboliser	-9.854	-21.040, 1.331	0.0850
	Intermediate Metaboliser	-0.423	-6.353, 5.507	0.889
	Ultrarapid Metaboliser	2.382	-19.155, 23.919	0.829
CYP1A2 [ref = *1/*1]	*1/*30	-0.084	-10.540, 10.371	0.987
	*30/*30	-5.278	-15.681, 5.125	0.321
CYP3A4 [ref = NM]	Reduced Metaboliser	-5.150	-18.343, 8.043	0.445
Age [continuous]		-0.230	-0.446, -0.015	0.0370
Sex [ref = Female]	Male	7.529	1.439, 13.619	0.0159
Ethnicity [ref = White]	Asian or Asian British	-4.825	-13.260, 3.609	0.263
	Black, Black British, Caribbean or African	-0.829	-11.196, 9.539	0.876
	Mixed or multiple ethnic groups	-10.456	-24.079, 3.168	0.133
	Other ethnic group	-7.185	-22.167, 7.797	0.348
Diagnosis [ref = schizophrenia]	Bipolar Disorder	-6.297	-16.174, 3.580	0.212
	Other psychotic disorder	-3.546	-11.226, 4.135	0.366
Antipsychotic medication [ref = aripiprazole]	Clozapine	-22.844	-33.447, -12.242	0.0000303
	Olanzapine	5.433	-4.621, 15.486	0.290
	Quetiapine	-12.065	-21.882, -2.248	0.0165
	Other antipsychotic (monotherapy)	-24.360	-33.509, -15.211	0.000000299
	Multiple antipsychotics	12.556	1.195, 23.918	0.0309
CYP3A4 inducer [ref = no]	Yes	9.588	-15.713, 34.889	0.458
CYP3A4 inhibitor [ref = no]	Yes	4.900	-7.816, 17.616	0.451
CYP1A2 inducer [ref = no]	Yes	6.775	-0.328, 13.879	0.0623
Antidepressant dose [continuous]		0.247	-6.909, 7.403	0.946
Mood stabiliser dose [continuous]		9.708	-1.666, 21.082	0.0952

Abbreviations: CI = confidence interval; NM = normal metaboliser; ref = reference category

Table S24. Liverpool University Neuroleptics Side Effects Rating Scale (LUNSERS) Items

LUNSERS Category	Side Effect Items
Extra Pyramidal	Muscle stiffness<br>Slowing of movements<br>Muscle spasms<br>Restlessness<br>Shakiness<br>Parts of the body moving of their own accord e.g. foot moving up and down<br>Over-wet or drooling mouth
Anticholinergic	Dry mouth<br>Constipation<br>Difficulty passing water<br>Blurred vision<br>Passing a lot of water
Other Autonomic	Dizziness<br>Feeling sick<br>Palpitations<br>Increased sweating<br>Diarrhoea
Allergic Reactions	Rash<br>Sensitivity to sun<br>New or unusual skin marks<br>Itchy skin
Psychic	Difficulty staying awake during the day<br>Increased dreaming<br>Difficulty in concentrating<br>Tension<br>Tiredness<br>Difficulty in remembering things<br>Lack of emotions<br>Depression<br>Sleeping too much<br>Difficulty getting to sleep
Hormonal	Swollen or tender chest<br>Period problems<br>Increased sex drive<br>Difficulty in achieving climax<br>Reduced sex drive<br>Periods less frequent
Miscellaneous	Headaches<br>Losing weight<br>Putting on weight<br>Pins and needles
Red Herrings	Runny nose<br>Chilblains<br>Hair Loss<br>Urine darker than usual<br>Weak fingernails<br>Mouth ulcers<br>Greasy skin<br>Flushing of face<br>Neck muscles aching<br>Painful joints

Table S25. Maximum British National Formulary (BNF) Doses of Antipsychotic Medications

Medication	Daily Maximum	Depot Maximum
Amisulpride	1200 mg / day	-
Aripiprazole	30 mg / day	400 mg / month
Cariprazine	6 mg / day	-
Clozapine	900 mg / day	-
Flupentixol	18 mg / day	400 mg / week
Haloperidol	20 mg / day	300 mg / month
Lurasidone	148 mg / day	300 mg / four weeks
Olanzapine	20 mg / day	300 mg / two weeks
Quetiapine	750 mg / day	-
Paliperidone	12 mg / day	150 mg / month
Promazine	800 mg / day	-
Risperidone	16 mg / day	100 mg / 28 days
Zuclopenthixol	150 mg / day	600 mg / week

Abbreviations: mg = milligrams
Note: The British National Formulary (BNF) percentage refers to the dose taken as a proportion of the maximum licensed dose under United Kingdom (UK) guidelines

Table S26. Maximum British National Formulary (BNF) Doses of Antidepressant Medications

Medication	Daily Maximum
Agomelatine	50 mg / day
Amitriptyline	150 mg / day
Citalopram	40 mg / day
Clomipramine	250 mg / day
Dosulepin	150 mg / day
Duloxetine	120 mg / day
Escitalopram	20 mg / day
Fluoxetine	60 mg / day
Imipramine	200 mg / day
Lofepramine	210 mg / day
Mirtazapine	45 mg / day
Paroxetine	50 mg / day
Sertraline	200 mg / day
Trazodone	300 mg / day
Venlafaxine	375 mg / day
Vortioxetine	20 mg / day

Abbreviations: mg = milligrams
Note: The British National Formulary (BNF) percentage refers to the dose taken as a proportion of the maximum licensed dose under United Kingdom (UK) guidelines

Table S27. Maximum British National Formulary (BNF) Doses of Mood Stabiliser Medications

Medication	Daily Maximum
Carbamazepine	1600 mg / day
Lamotrigine	400 mg / day
Lithium carbonate	1200 mg / day*
Sodium valproate	2500 mg / day

* No specified maximum daily dose is given by the BNF for lithium, so maximum dose is taken from other sources, including the Health Service Executive (Ireland)
Abbreviations: mg = milligrams
Note: The British National Formulary (BNF) percentage refers to the dose taken as a proportion of the maximum licensed dose under United Kingdom (UK) guidelines

Table S28. Descriptive Statistics for All Covariates Used in Regression Models

Variable	Full Sample	Psychosis Diagnosis-Only Subgroup
**Age**, mean years (SD)	43.5 (14.5)	43.5 (14.4)
Age range, years	18 – 82	18 – 82
**Sex**, *n* (%)		
Male	245 (54.1%)	22 6 (56.5%)
Female	208 (45.9%)	174 (43.5%)
**Ethnicity**, *n* (%)		
Asian or Asian British	67 (14.8%)	59 (14.8%)
Black, Black British, Caribbean or African	44 (9.7%)	44 (11.0%)
White	302 (66.7%)	259 (64.8%)
Mixed or multiple ethnic groups	21 (4.6%)	20 (5.0%)
Other ethnic group	19 (4.2%)	18 (4.5%)
**Diagnosis**, *n* (%)		
Schizophrenia	164 (36.2%)	164 (41.0%)
Bipolar disorder	110 (24.3%)	110 (27.5%)
Other psychotic disorder	126 (27.8%)	126 (31.5%)
Other psychiatric disorder	53 (11.7%)	-
**Antipsychotic medication**, *n* (%)		
Aripiprazole	85 (18.8%)	74 (18.5%)
Clozapine	44 (9.7%)	44 (11.0%)
Olanzapine	66 (14.6%)	61 (15.2%)
Quetiapine	74 (16.3%)	46 (11.5%)
Other antipsychotic medication	103 (22.7%)	97 (24.2%)
2+ different antipsychotic medications	81 (17.9%)	78 (19.5%)
**Antipsychotic dose**, mean BNF percentage (SD)	51.2 (33.3)	53.5 (33.3)
Antipsychotic dose range, BNF percentage	1.25 – 166.7	1.25 – 166.7
**Antidepressant dose**, mean BNF percentage (SD)	31.2 (45.5)	26.7 (43.3)
Antidepressant dose range, BNF percentage	0 – 233.3	0 – 233.3
**Mood stabiliser dose**, mean BNF percentage (SD)	16.6 (32.9)	17.9 (34.1)
Mood stabiliser BNF range, percentage	0 – 248.3	0 – 248.3
**Taking a CYP1A2 inducer**, *n* (%)	112 (24.7%)	102 (25.5%)
**Taking a CYP3A4 inducer**, *n* (%)	9 (2.0%)	8 (2.0%)
**Taking a CYP3A4 inhibitor**, *n* (%)	29 (6.4%)	25 (6.2%)

Abbreviations: SD = standard deviation; n = number of participants; BNF = British National Formulary

Note: The British National Formulary (BNF) percentage refers to the dose taken as a proportion of the maximum licensed dose under United Kingdom (UK) guidelines
